# Functional Suppression of SCAP Triggers Endoplasmic Reticulum Stress‐Dependent Ferroptosis by Impairing Cholesterol Metabolism in Gastric Cancer

**DOI:** 10.1002/advs.76290

**Published:** 2026-06-29

**Authors:** Qianqian Xu, Guangzhao Pan, Lele Zhang, Xiangliu Chen, Kui Zhang, Yang Lu, Zibo Duan, Aiping Chen, Hailong Shen, Yuxi Zhang, Xiaowu Dong, Jinzhou Huang, Xing Huang, Kai Miao, Qian Hua, Fangfang Tao, Weidong Zhang, Jiang‐Jiang Qin

**Affiliations:** ^1^ Center for Innovative Drug Research Hangzhou Institute of Medicine (HIM) Chinese Academy of Sciences Hangzhou Zhejiang China; ^2^ Institute of Medicinal Plant Development Chinese Academy of Medical Sciences and Peking Union Medical College Beijing China; ^3^ Zhejiang Cancer Hospital, Hangzhou Institute of Medicine (HIM) Chinese Academy of Sciences Hangzhou Zhejiang China; ^4^ School of Basic Medical Sciences Zhejiang Chinese Medical University Hangzhou Zhejiang China; ^5^ School of Preclinical Medicine Chengdu University Chengdu China; ^6^ Pritzker School of Molecular Engineering Ben May Department for Cancer Research University of Chicago Chicago USA; ^7^ Hangzhou Institute of Innovative Medicine, Institute of Drug Discovery and Design, College of Pharmaceutical Sciences Zhejiang University Hangzhou Zhejiang China; ^8^ School of Life Sciences Beijing University of Chinese medicine Beijing China; ^9^ Zhejiang Provincial Key Laboratory of Pancreatic Disease, The First Affiliated Hospital Zhejiang University School of Medicine Hangzhou Zhejiang China; ^10^ MOE Frontier Science Centre for Precision Oncology University of Macau Macau SAR China; ^11^ Zhejiang Key Laboratory of Blood‐Stasis‐Toxin Syndrome, Zhejiang Chinese Medical University China Collaborative Graduate School of Traditional Chinese Medicine Hangzhou Zhejiang China; ^12^ Department of Phytochemistry School of Pharmacy Second Military Medical University Shanghai China

**Keywords:** cholesterol metabolism, endoplasmic reticulum stress, ferroptosis, gastric cancer, SCAP, sterol‐sensing domain

## Abstract

Cholesterol metabolic reprogramming is an emerging vulnerability in cancer, yet clinical progress has been limited by a lack of druggable targets. Here, we identify the sterol‐sensing domain (SSD) of SCAP as a target in gastric cancer (GC), with multi‐omics confirming tumor‐specific SCAP overexpression, poor prognosis, and hyperactivated synthesis. Using SSD structure‐based high‐throughput screening, we discovered that the natural compound Platycodin D (PD) is a small‐molecule inhibitor of SCAP. PD sustains SREBP2 activation yet paradoxically blocks cholesterol efflux. The underlying mechanism is that PD specifically disrupts SCAP's sterol‐sensing function, thereby permitting unrestrained SREBP2‐mediated biosynthesis. This critical dysfunction leads to pathological cholesterol overload in the endoplasmic reticulum (ER), inducing proteotoxic stress. Consequently, this stress disrupts Nrf1 ER retention and forces nuclear translocation, thereby compromising LXR‐mediated efflux. We further demonstrate that this SCAP targeting initiates GPX4 cascade‐mediated ferroptosis, which was reversible by inhibiting cholesterol synthesis or the stress response. PD demonstrated potent tumor suppression with a significantly improved safety profile compared to cisplatin in vivo. Our work establishes a causal link between SSD disruption and ferroptotic death via cholesterol dysregulation, introducing a novel paradigm for exploiting metabolic vulnerabilities in GC therapy.

## Graphical abstract

This study reveals a novel GC therapy targeting cholesterol homeostasis. Inhibiting the SCAP sterol‐sensing domain causes lethal ER stress via cholesterol accumulation, paradoxically forcing continuous synthesis that triggers ferroptosis. The findings link sterol sensing, metabolic dysregulation, and ferroptosis, establishing an anti‐cancer paradigm. The identified natural compound offers a safe, potent alternative to cancer therapy, providing a framework for targeting cholesterol metabolism across GC.

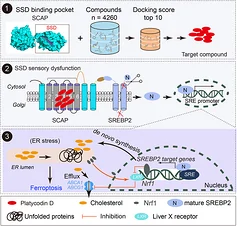

## 1 Background

Gastric cancer (GC), the fifth most commonly diagnosed malignancy and the third leading cause of global cancer mortality, continues to present significant therapeutic challenges, particularly in the development of effective targeted therapies [1]. Metabolic reprogramming has emerged as a key hallmark of cancer, strongly associated with tumor initiation and progression [2]. Among the diverse metabolic alterations in cancer cells, dysregulation of cholesterol homeostasis stands out as one of the most prominent and functionally significant features [2]. Cholesterol serves not only as an essential structural component of membranes but also a signaling molecule that supports oncogenesis via tightly regulated biosynthesis and metabolism pathways, thereby driving sustained proliferation, signal transduction, tumor microenvironment remodeling, and therapy resistance [3, 4, 5]. Cholesterol interacts with other metabolic pathways to exert important effects on cell fate determination. Cholesterol metabolism influences the proliferation, differentiation, and migration of stem cells through multiple pathways, including AKT/FOXO1, Notch, and AKT/ERK [6, 7]. Moreover, dysregulated cholesterol metabolism is not merely a consequence of aging‑associated diseases but has been demonstrated to directly induce cellular senescence [8, 9]. Notably, recent studies have revealed a close relationship between cholesterol and ferroptosis. In the terminal steps of cholesterol biosynthesis, the distal metabolite 7‑dehydrocholesterol (7‑DHC) acts as a ferroptosis suppressor [10]. Unexpectedly, recent studies also found that excessive cholesterol accumulation can promote cell death [11, 12], suggesting that cholesterol homeostasis is a key factor in maintaining normal cellular physiology. Therefore, targeting cholesterol metabolism represents a promising therapeutic strategy for GC.

Cellular cholesterol homeostasis is dynamically maintained through a balance of de novo biosynthesis, lipoprotein‐mediated uptake, efflux, and esterification [13]. Under physiological conditions, cholesterol levels are tightly controlled by feedback mechanisms involving sterol‐responsive transcription factors and metabolic sensors. Sterol regulatory element‐binding proteins (SREBPs) act as master regulators, with three endoplasmic reticulum (ER)‐anchored isoforms serving distinct roles: SREBP‐1a activates broad lipogenic programs, SREBP‐1c preferentially enhances fatty acid synthesis, and SREBP‐2 specifically governs cholesterol biosynthesis and low density lipoprotein (LDL) receptor expression [14, 15]. All SREBP isoforms require interaction with SREBP cleavage‐activating protein (SCAP), an ER‐resident cholesterol sensor containing a sterol‐sensing domain (SSD), for proteolytic activation and nuclear translocation [14, 16]. SCAP thus functions as the central rheostat linking cellular sterol levels to SREBP‐mediated transcriptional responses.

The 1,276‐amino acid SCAP protein contains five structurally defined domains that collectively regulate cholesterol homeostasis. Its SSD (residues 284–422), formed by transmembrane helices S2–S6 within Loop1, encompasses a cholesterol‐binding pocket essential for sterol detection. The INSIG‐binding domain facilitates sterol‐dependent regulatory interactions, while the MELADL motif in Loop6 mediates COPII vesicle loading via Sec24 binding. Loop7 contributes to structural stability, and the C‐terminal WD40 repeat domain facilitates SREBP2 complex assembly. Functioning as a sterol‐responsive biosensor, SCAP controls SREBP2 translocation from the ER to the Golgi, thereby modulating cholesterol biosynthetic gene expression [16, 17, 18, 19]. Emerging therapeutic strategies targeting this axis include: blocking ER‐Golgi translocation by disrupting Sec24‐MELADL interaction [20, 21]; inhibiting Golgi processing via glycosylation interference [22, 23]; promoting ubiquitin‐proteasome‐mediated SCAP degradation [24]; suppressing SREBP transcription [25]; dissociating the COPII coatomer complex [26]; and attenuating lipid kinase signaling [27, 28]. Although several SCAP inhibitors show efficacy [24], the SSD remains an underexploited therapeutic target. We hypothesized that targeting the SSD with small molecules could disrupt sterol sensing and exert antineoplastic effects by dysregulating cholesterol metabolism.

In this study, we demonstrate that SCAP is overexpressed in GC tissues, correlating with elevated cholesterol levels and poor prognosis, underscoring its clinical relevance. We identify a novel functional SCAP inhibitor targeting the SSD that activates de novo cholesterol synthesis without degrading SCAP, leading to pathological ER cholesterol overload. Mechanistically, this inhibitor disrupts nuclear factor erythroid 2‐related factor 1 (Nrf1/NFE2L1), an ER‐resident Cap'n’Collar transcription factor and established cholesterol sensor [29], triggering its proteolytic activation and nuclear translocation. Consequently, impaired LXR‐mediated efflux overrides homeostatic feedback, resulting in a synthetic lethality outcome: extreme ER cholesterol overload induces ferroptosis in cancer cells. This vulnerability was confirmed in patient‐derived xenograft and organoid models, showing that SSD inhibition disrupts cholesterol homeostasis via sustained SREBP signaling and disabled Nrf1‐mediated efflux. Together, our findings establish cholesterol homeostasis as a targetable vulnerability in cancer cells, offering new insights for therapeutic development in GC.

## 2 Results

### 2.1 SCAP Exhibits Tumor‐Specific Overexpression and Serves as a Clinically Relevant Prognostic Biomarker in GC Patients

**FIGURE 1:**
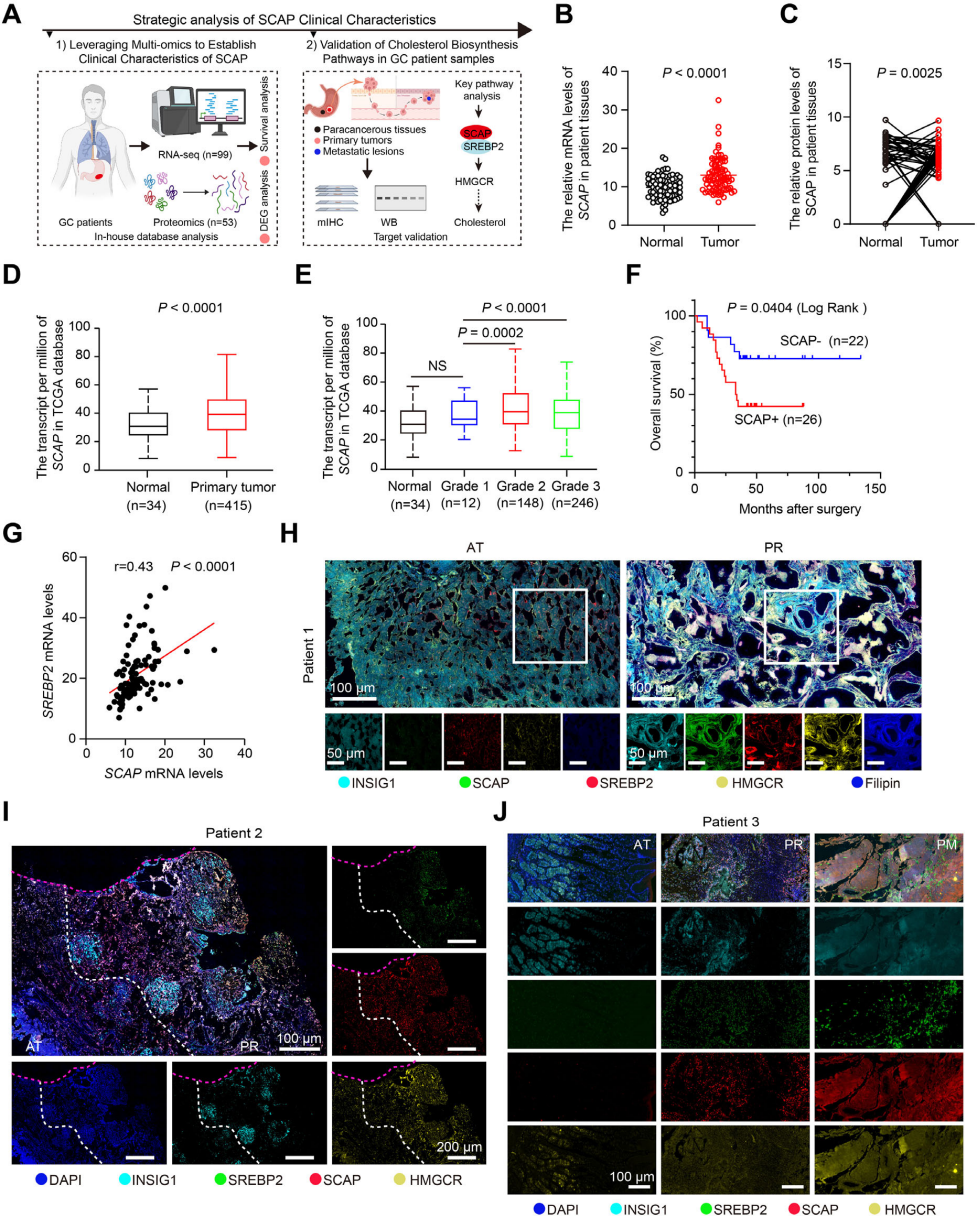
SCAP is overexpressed in GC and correlates with poor prognosis. (A) Workflow for analyzing SCAP clinical characteristics in GC patients. The strategy included two main components: (1) assessing SCAP's clinical relevance using an in‐house database, including tissue expression differences and patient prognosis; (2) pathological analysis of 15 paired tumor tissues, normal adjacent tissues, and peritoneal metastatic foci, focusing on cholesterol levels and key regulators of cholesterol synthesis. (B, C) SCAP mRNA expression (B) in paired GC samples (*n* = 75) and SCAP protein levels (C) in paired GC tissues (*n* = 53) from the in‐house database. (D) *SCAP* mRNA levels in normal (*n* = 34) and GC tissues (*n* = 415) using The Cancer Genome Atlas (TCGA). (E) *SCAP* mRNA expression across tumor grades in GC (TCGA). Normal (*n* = 34); Grade 1 (*n* = 12); Grade 2 (*n* = 148); Grade 3 (*n* = 246). (F) Correlation between SCAP expression and patient survival in the in‐house GC cohort. (G) Correlation analysis of *SCAP* and *SREBP2* mRNA levels using TCGA database. (H, I) Representative multiplex immunohistochemical images showing SCAP, SREBP2, HMGCR, INSIG1, and Filipin staining in paired GC tissues from Patient 1 and Patient 2. Scale bar = 100 µm (H) and 200 µm (I). AT, normal adjacent tissues; PR, primary tumor tissues. (J) Representative images (Patient 3) showing expression of SCAP, SREBP2, HMGCR, and INSIG1 across different GC pathological subtypes. Scale bar = 100 µm.

‌Initial evaluation of SCAP's clinical characteristics supported its potential as a therapeutic target (Figure 1A). Analysis of 75 paired GC specimens revealed significantly higher SCAP mRNA and protein levels in tumor tissues compared with adjacent normal tissues (Figure 1B,C), a finding corroborated by TCGA data (Figure 1D) and further reflected by a grade‐dependent increase in SCAP expression (Figure 1E). In our cohort of 48 surgically resected GC patients (from an in‐house database), stratification based on median SCAP expression showed that patients with high SCAP expression (SCAP+, *n* = 26) had significantly shorter overall survival than those with low SCAP expression (SCAP‐, *n* = 22; *p* = 0.0404; Figure 1F). We next assessed the expression of sterol regulatory element‐binding protein 2 (SREBP2), a key transcriptional regulator of cholesterol biosynthesis [13], to evaluate cholesterol pathway alterations during GC progression. *SREBP2* mRNA expression was upregulated in tumor tissues and exhibited a positive correlation with SCAP mRNA levels (Figure S1A; Figure 1G).

‌To validate these observations, we profiled cholesterol metabolism and de novo synthesis pathways in clinical specimens from 15 additional GC patients. We measured cholesterol levels and key regulatory proteins (SCAP, INSIG1, SREBP2, and HMGCR) [13]. Multiplex immunohistochemistry (mIHC) revealed marked upregulation of filipin‐stained cholesterol deposits and cholesterol synthesis‐related proteins in primary tumor (PR) compared to normal adjacent tissues (AT) (Figure 1H–J; Figure S1B–E). These findings were further confirmed by western blot analysis in independent patient cohorts (Figure S1F). Moreover, protein expression levels increased progressively with advancing disease stage, from AT to peritoneal metastases (PM) (Figure 1J; Figure S1D,E), suggesting activation of adaptive metabolic reprogramming pathways to meet rising cholesterol demands during tumor progression. Taken together, these results underscore a dysregulation of cholesterol signaling in GC and identify SCAP, SREBP2, and HMGCR as clinically relevant biomarkers for assessing malignant progression.

### 2.2 High‐Throughput Screening for Potential Function Inhibitors of SCAP SSD

**FIGURE 2:**
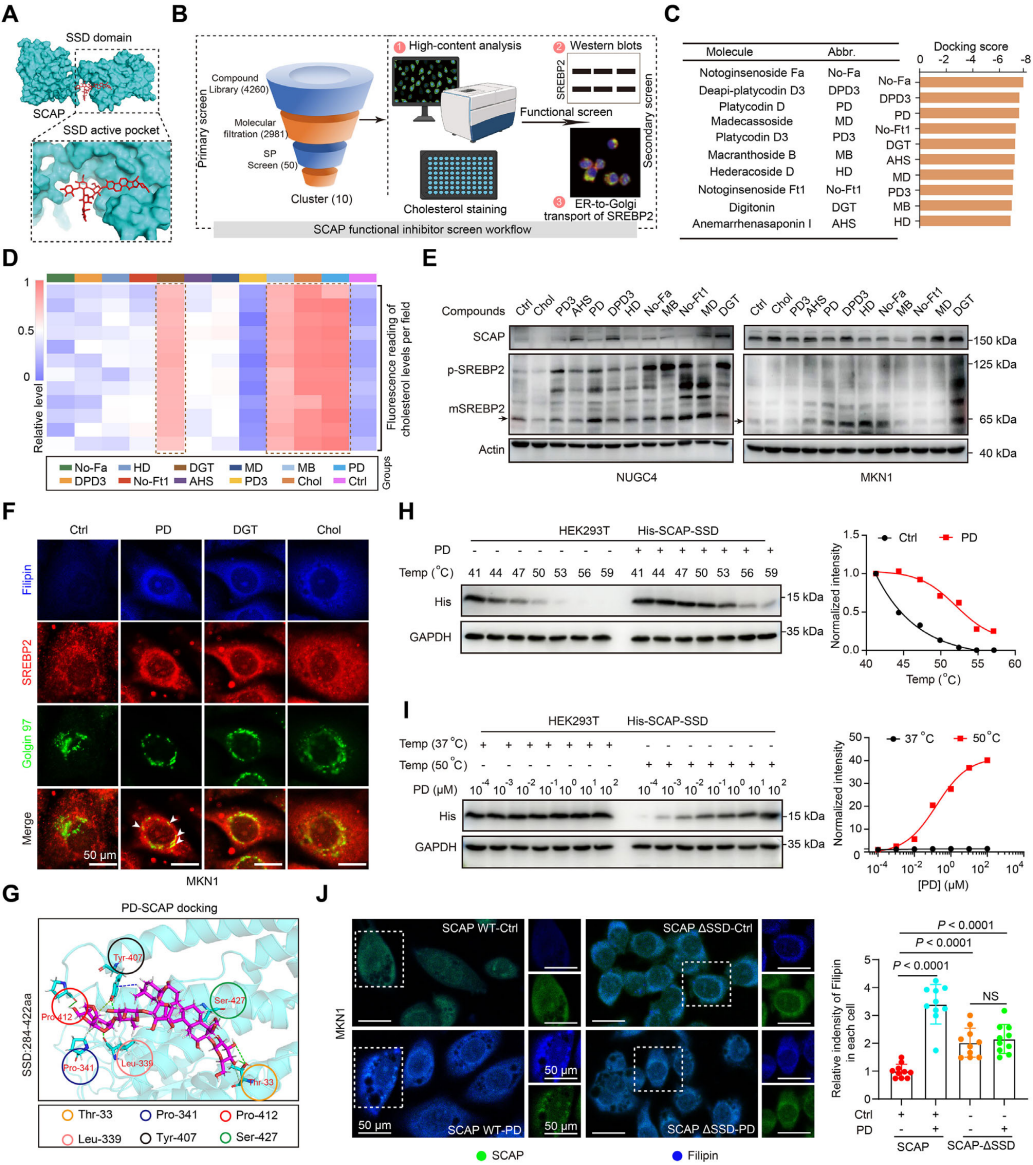
PD is identified as a functional inhibitor of the SCAP sterol‐sensing domain. (A) Schematic of the SCAP sterol‐sensing domain (SSD) active pocket. (B) Workflow for screening functional SCAP SSD inhibitors. Primary screening involved virtual docking of the SCAP‐SSD against a 4260‐compound drug library. Functional screening combined high‐content imaging, immunoblotting, and SREBP2 ER‐to‐Golgi transport assays. ER, endoplasmic reticulum. (C) Top 10 SCAP SSD‐interacting compounds ranked by docking score. (D) Changes in cellular cholesterol levels induced by the top 10 candidate compounds, measured by high‐content imaging. Ctrl, control; Chol, cholesterol. (E) Immunoblotting analysis of SCAP, precursor SREBP2 (pSREBP2), and mature SREBP2 (mSREBP2) in NUGC4 and MKN1 cells treated with the top 10 compounds for 24 h. (F) Representative immunofluorescence images showing SREBP2 colocalization with the Golgi (marked by Golgin 97) and intracellular cholesterol levels (filipin staining) in cells treated with PD, DGT, or Chol. Scale bar = 50 µm. (G) Kinetic simulation of the interaction between the SCAP SSD (cyan) and PD (purple) at 200 ns. The SCAP protein (PDB ID: 7ETW) crystal structure file was downloaded from RCSB. (H, I) Immunoblots of HEK293T cells stably expressing His‐SCAP SSD (aa 284–422) treated with 20 µm PD at different temperatures (H) or with increasing PD concentrations at 37°C or 50°C (I). Graphs show quantified protein levels. (J) Representative images of Filipin‐stained cholesterol in SCAP knockdown MKN1 cells reconstituted with SCAP‐WT or SCAP‐ΔSSD (lacking aa 284–442), treated with vehicle or 10 µm PD for 24 h. Scale bar = 50 µm. NS, not significant.

SCAP interacts with INSIG1/2 via its SSD in response to cholesterol levels, thereby modulating transcription of cholesterol synthesis and uptake genes [30]. Targeting the SSD with small molecules represents a promising strategy to disrupt cellular cholesterol sensing, inhibit cholesterol synthesis and metabolism, and ultimately suppress tumor growth. We therefore selected the SSD as a putative drug‐binding pocket and conducted multiple rounds of virtual and functional screenings to identify functional inhibitors (Figure 2A,B). Given that natural product‐derived fatty acids often exhibit high conformational flexibility, frequently resulting in low‐activity compounds with excessive rotatable bonds, we pre‐filtered our compound libraries to retain only molecules with ≤ 12 rotatable bonds (*n* = 2981). Structure‐based virtual screening using glide (SP mode) was then performed, and the top‐scoring compounds (docking score ≤ ‐7) were selected. From these, the 50 highest‐ranked molecules underwent clustering analysis, and ten candidates were manually chosen for experimental validation based on binding pose analysis and chemical diversity (Figure 2B,C).

Cellular cholesterol homeostasis is maintained through feedback regulation between synthesis and efflux pathways. Dysfunctional cholesterol sensing disrupts this balance, leading to sustained activation or suppression of cholesterol synthesis, providing a rationale for targeted therapeutic screening [14]. We next performed secondary screening of candidate compounds using high‐content screening (HCS), western blot, and immunofluorescence (IF) assays (Figure S2A,B; Figure 2D–F). We aimed to identify inhibitors that induce sustained activation of the cholesterol synthesis pathway, as assessed by mature SREBP2 (mSREBP2) and cholesterol levels. Filipin‐based HCS revealed that platycodin D (PD), macranthoside B (MB), and digitonin (DGT) potently induced cellular cholesterol accumulation (Figure 2D; Figure S2A,B).

Mechanistically, under low‐cholesterol conditions, SCAP escorts SREBP2 via COPII vesicles to the Golgi, where Site‐1 protease (S1P) and Site‐2 protease (S2P) sequentially cleave SREBP2 to generate the mature N‐terminal fragment mSREBP2. The mSREBP2 fragment translocates to the nucleus and activates sterol‐responsive genes by binding to sterol regulatory elements (SREs), making mSREBP2 detection a specific indicator of pathway activation [16, 30]. Immunoblot analysis revealed cell line‐dependent effects: in NUGC4 cells, anemarrhenasaponin I (AHS), PD, deapi‐platycodin D3 (DPD3), hederacoside D (HD), notoginsenoside Fa (No‐Fa), and DGT elevated mSREBP2 levels; in MKN1 cells, PD, No‐Fa, MB, notoginsenoside Ft1 (No‐Ft1), madecassoside (MD), and DGT showed similar activity (Figure 2E), indicating that genetic background influences compound efficacy. Notably, cholesterol treatment suppressed SREBP2 maturation, while PD‐mediated SREBP2 activation was the most pronounced in both cell lines (Figure 2E). Colocalization assays using the Golgi marker Golgin 97 confirmed that PD and DGT significantly promoted SREBP2 trafficking to the Golgi (Figure 2F;Figure S2B).

Consistent with prior structural studies, Yan et al. demonstrated that DGT acts as an effective molecular detergent that stabilizes the SCAP/INSIG‐2 complex conformation within molecular micelles [30, 31]. Importantly, DGT occupies the same binding pocket in SCAP/INSIG‐2 complexes as 25 hydroxycholesterol (25‐HC), revealing evolutionary conservation of sterol‐sensing mechanisms. These results validate the robustness of our screening strategy and underscore the potential of the SSD pocket as a druggable target worthy of systematic interrogation.

### 2.3 Identification of PD as a Novel Specific Inhibitor Targeting the SSD of SCAP

To further investigate the interaction between PD and SCAP, we conducted molecular dynamics simulations. The results demonstrated that the PD‐SCAP complex is stabilized by multiple intermolecular forces, including five hydrogen bonds (Thr‐33×2, Tyr‐40, Leu‐339, Pro‐412), five C–H interactions (Thr‐33, Pro‐341, Tyr‐407, Ser‐427×2), one Pi‐Sigma interaction (Tyr‐401), and six hydrophobic interactions (Leu‐339, Leu‐419, Val‐422, Val‐423, Val‐426×2) (Figure S2C). Notably, these interactions were largely confined to the SCAP SSD (284‐442aa) (Figure 2G), indicating that PD binds specifically to the SSD and contributes to complex stabilization. In addition to the interaction forces provided by PD, several key residues within SCAP were found to play a major role in mediating these contacts (Figure S2D), collectively forming a binding pocket that supports specific molecular recognition.

The root mean square deviation (RMSD) of the PD‐SCAP complex stabilized after 150 ns of binding, with only minor fluctuations within a limited spatial range, indicating system equilibrium and convergence (Figure S2E). Radius of gyration (*R*_g_) analysis showed a gradual decrease over the simulation time, suggesting that PD binding induces a more compact and stable conformation of the complex (Figure S2F). Furthermore, Gibbs free energy analysis confirmed that the SCAP‐PD interaction is thermodynamically favorable (Figure S2G).

We next employed cellular thermal shift assays (CETSA) to investigate the effect of PD on the thermal stability of SCAP. PD treatment enhanced the thermal stability of full‐length SCAP (Figure S2H) and exerted temperature‐ and dose‐dependent stabilizing effects specifically on the SSD (Figure 2H,I). To functionally validate these findings, we expressed an SSD‐deficient SCAP mutant in SCAP‐knockdown (KD) tumor cells and assessed the impact of PD on cholesterol synthesis. IF experiments revealed that SSD deletion not only significantly increased intracellular cholesterol levels (*p* < 0.0001) but also abolished PD's ability to induce cholesterol accumulation (Figure 2J). Taken together, these findings demonstrate that PD acts as a selective inhibitor targeting the SSD of SCAP.

### 2.4 PD Induces Cholesterol Metabolic Dysregulation by Remodeling De Novo Biosynthesis and Efflux

**FIGURE 3:**
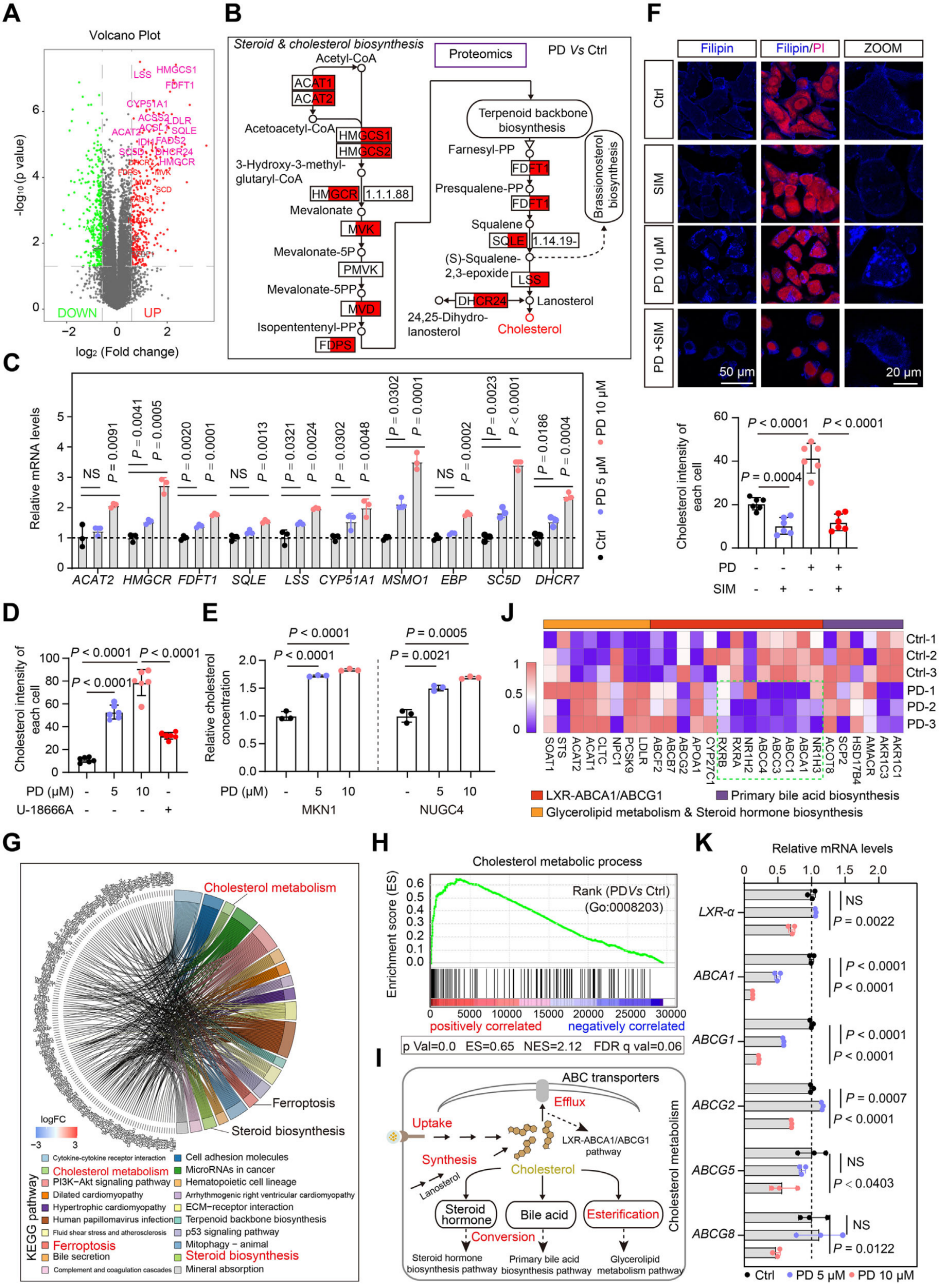
PD impairs cholesterol metabolism by dysregulating de novo biosynthesis and efflux. (A) Volcano plot of differentially expressed proteins in PD‐treated versus vehicle‐treated cells (*n* = 3 replicates per group). Pink dots denote proteins involved in cholesterol synthesis; gray dots indicate non‐significant changes. UP, upregulated; DOWN, downregulated. (B) Pathway analysis of steroid/cholesterol biosynthesis based on proteomics, showing protein enrichment in PD versus control cells. Rectangles represent protein levels; red, upregulated; gray, unchanged (*n* = 3 cell replicates per group). (C) Relative mRNA levels of steroid/cholesterol biosynthesis genes in vehicle‐ and PD‐treated MKN1 cells (*n* = 3 biological replicates). (D) Intracellular cholesterol levels (filipin staining) in cells treated with vehicle, PD (5 or 10 µm), or U‐18666A. U‐18666A serves as a cholesterol transport inhibitor (see Figure S3E for additional information). (E) Intracellular total cholesterol levels in MKN1 and NUGC4 cells treated for 24 h with vehicle or PD (5 or 10 µm), measured using the Amplex Red Cholesterol Assay Kit (*n* = 3 biological replicates). (F) Intracellular cholesterol levels in cells treated with vehicle, PD alone, or PD combined with simvastatin (SIM), an HMGCR inhibitor. (G) Pathway enrichment profiles comparing vehicle and PD groups (proteomics). (H) GSEA of cholesterol metabolic process in vehicle versus PD groups (RNA‐seq). (I) Schematic of cholesterol metabolism in tumor cells. (J) Heatmap of differentially expressed proteins in the cholesterol metabolism pathway from vehicle versus PD groups (proteomics). (K) Relative mRNA levels of cholesterol efflux genes in vehicle‐ and PD‐treated MKN1 cells (*n* = 3 biological replicates). NS, not significant.

Although previous studies have reported functional inhibition of SCAP [24, 32, 33], the physiological consequences of targeting the SCAP SSD remain unclear. To explore PD's functional impact via SSD targeting, we performed integrated multi‐omics analyses. Proteomic and transcriptomic profiling after PD treatment revealed significant upregulation of sterol/cholesterol biosynthetic pathways and key enzymes, including HMGCR, FDFT1, CYP51A1, LSS, and SQLE (Figure 3A;Figure S3A–C). De novo cholesterol synthesis begins with acetyl‐CoA and proceeds through HMGCR‐catalyzed mevalonate production, culminating in cholesterol via multi‐step enzymatic catalysis (Figure 3B;Figure S3B). Using RT‐qPCR, we further validated pronounced upregulation of mRNA expression for enzymes across the synthesis cascade (*HMGCR*, *FDFT1*, *SQLE*, *LSS*, *CYP51A1*, *MSMO1*, *EBP*, *SC5D*, and *DHCR7*) (Figure 3C;Figure S3D). Pathway enrichment analysis also highlighted significant activation of cholesterol synthesis and related processes upon PD treatment, underscoring the robustness of our screening (Figure S3C).

To distinguish de novo synthesis from serum‐derived cholesterol, cells were serum‐starved for 24 h before PD treatment [34]. PD‐treated cells still accumulated substantial cholesterol, even exceeding the levels induced by the cholesterol transport inhibitor U‐18666A (Figure 3D; Figure S3E). Meanwhile, PD treatment elevated total cellular cholesterol levels, as quantified by the Amplex Red assay (Figure 3E), which was reversed by co‐treatment with simvastatin (SIM), a potent HMGCR inhibitor (Figure 3F). These results demonstrate that pharmacological targeting of the SSD activates de novo cholesterol synthesis, and the resulting accumulation stems primarily from endogenous biosynthesis rather than exogenous uptake.

Notably, PD‐induced cholesterol accumulation reflects a disruption of sterol homeostasis, as endogenous feedback mechanisms normally suppress de novo cholesterol synthesis to maintain balance [35, 36]. Multi‐omics profiling showed concomitant activation of cholesterol synthesis and metabolic pathways (Figure 3G; Figure S3F,H). Paradoxically, although most metabolic genes were transcriptionally upregulated, functional metabolic activation did not flow (Figure 3H;Figure S3G). Instead, transcriptomic analysis indicated substantial lipid metabolism disorder (Figure S3G). In most cells, cholesterol homeostasis is maintained through cholesterol biosynthesis, uptake, efflux, conversion, and esterification (Figure 3I). While most pathways were activated or remained unchanged upon PD treatment, we observed significant suppression of genes involved in cholesterol efflux (indicated by the green dotted box, Figure 3J), suggesting impaired efflux as another key contributor to accumulation. Under physiological conditions, rising intracellular cholesterol promotes oxysterol formation, activating the Liver X receptors (LXRs) transcriptional network to stimulate cholesterol efflux while curtailing synthesis and uptake [37]. However, PD intervention suppressed the LXR pathway. RT‐qPCR confirmed downregulation of cholesterol efflux transporters (e.g., *ABCA1* and *ABCG1*) and the lipid‐activated transcription factors (e.g., *LXRα*) [38], indicating a broad imbalance in cholesterol metabolic regulation (Figure 3K; Figure S3I).

### 2.5 PD Targeting SSD Facilitates Golgi Translocation of SCAP‐SREBP2 Complex and SREBP2 Maturation

Whether PD binding to the SCAP SSD disrupts cellular cholesterol sensing remains to be determined. Under sterol‐depleted conditions, the hexapeptide motif in SCAP loop 6 facilitates the transport of the SCAP‐SREBP complex to the Golgi (Figure S4A, left), where the proteases Site‐1 (S1P) and Site‐2 (S2P) sequentially cleave SREBP, releasing its transcription factor domain for nuclear translocation and lipogenic activation. In contrast, under high sterol conditions, conformational changes in the SCAP L1 domain promote INSIG1/2 binding (Figure S4A, right), thereby retaining the complex in the ER and suppressing lipid biosynthesis [14].

**FIGURE 4:**
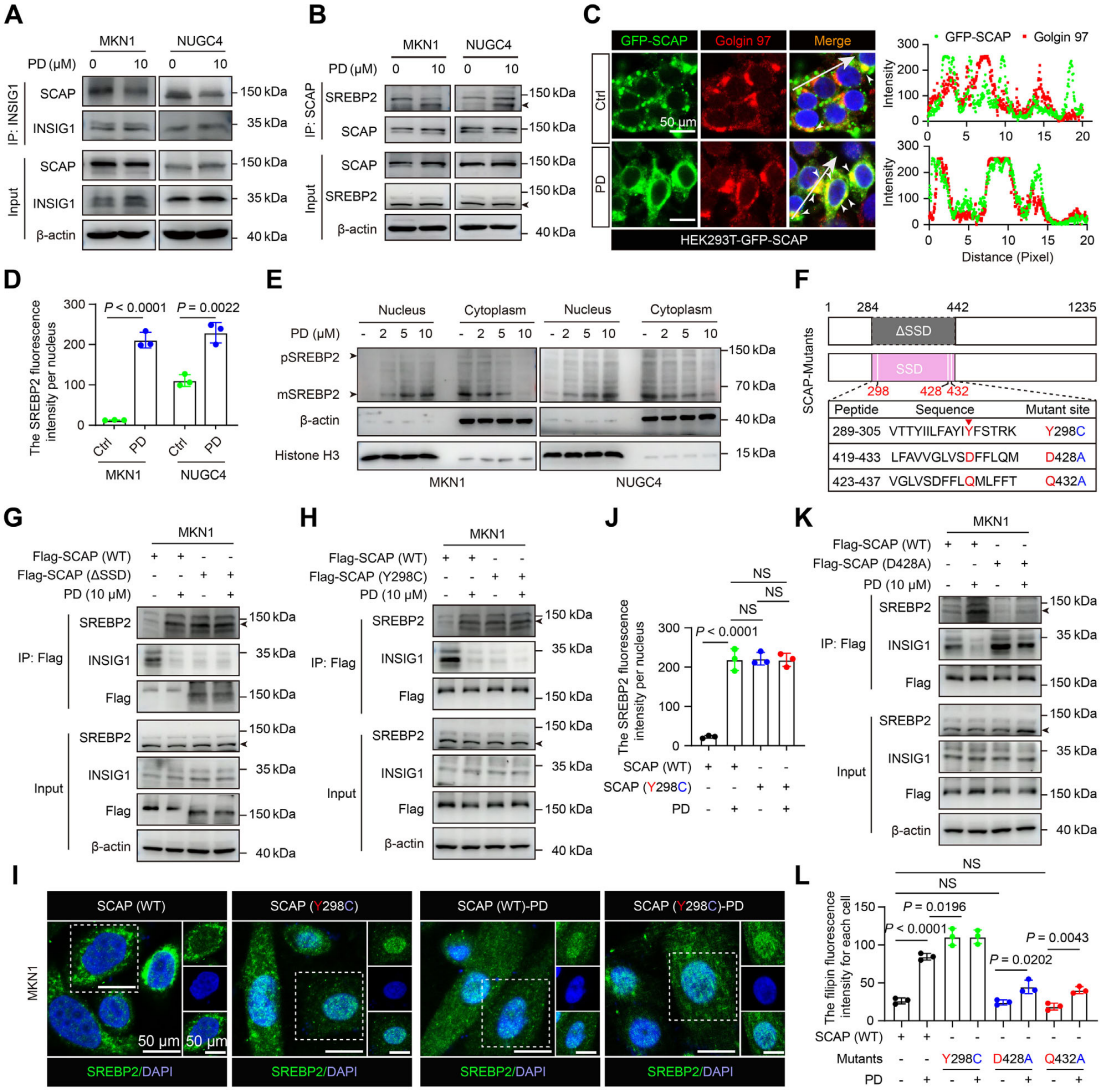
PD binding to SSD promotes Golgi trafficking of the SCAP‐SREBP2 complex and SREBP2 proteolytic activation. (A, B) Endogenous co‐immunoprecipitation of SCAP‐INSIG1 (A) and SCAP‐SREBP2 (B) complexes in MKN1 and NUGC4 cells. Cell lysates were immunoprecipitated with anti‐INSIG1 or anti‐SCAP antibody for 12 h, followed by immunoblotting. (C) Representative immunofluorescence images showing SCAP colocalization with the Golgi in HEK293T cells stably expressing GFP‐SCAP, treated with vehicle or 10 µm PD for 6 h. Scale bar = 50 µm. (D) Quantification of SREBP2 nuclear translocation intensity (see Figure S4C,D for additional information). (E) Nuclear and cytoplasmic SREBP2 levels in MKN1 and NUGC4 cells treated with PD, assessed by immunoblotting. (F) Bioinformatics prediction of mutant sites within the SCAP SSD. (G, H) Immunoblots of Flag, SREBP2, and INSIG1 in SCAP‐knockdown MKN1 cells reconstituted with Flag‐SCAP WT, ΔSSD (g) or Y298C (H) mutant. Cells were treated with PD and subjected to Flag immunoprecipitation. (I, J) Representative immunofluorescence images (I) and quantification (J) of SREBP2 nuclear translocation in SCAP‐knockdown MKN1 cells expressing Flag‐SCAP WT or Y298C mutant, treated with vehicle or 10 µM PD for 24 h. Scale bar = 50 µm. (K) Immunoblots of Flag, SREBP2, and INSIG1 in SCAP‐knockdown MKN1 cells reconstituted with Flag‐SCAP WT or D428A mutant after PD treatment and Flag immunoprecipitation. (L) Quantification of Filipin intensity in SCAP‐knockdown MKN1 cells expressing WT or mutant Flag‐SCAP (Y298C, D428A, Q432A), treated with vehicle or 10 µM PD for 24 h (see Figure S4H for additional information). WT, wild type; NS, not significant.

We next assessed the effect of PD on key regulatory proteins and found that it did not alter total SCAP or INSIG1 levels, but markedly increased mSREBP2 levels (Figure S4B). Given the critical role of SCAP‐INSIG1 and SCAP‐SREBP2 complexes in cholesterol sensing, we investigated how PD influences this process in MKN1 and NUGC4 cells. Co‐immunoprecipitation (Co‐IP) assays showed that PD treatment significantly attenuated the SCAP‐INSIG1 interaction while enhancing SCAP‐SREBP2 complex formation (Figure 4A,B). To directly visualize the trafficking of the SCAP‐SREBP2 complex, we performed IF staining, which revealed increased colocalization of SCAP with the Golgi marker Golgin 97 upon PD treatment in HEK293T cells (Figure 4C). More importantly, PD enhanced nuclear translocation of SREBP2 in a dose‐dependent manner, as confirmed by SREBP2‐DAPI colocalization assays and nucleocytoplasmic separation experiments (Figure 4D,E; Figure S4C,D). Together, these results demonstrate that PD promotes cholesterol biosynthesis by facilitating SREBP2 proteolytic maturation, representing the primary mechanism for activation of de novo synthesis.

### 2.6 Tyrosine 298 of SCAP SSD Is Essential for PD‐Mediated SREBP2 Activation

To further confirm that PD activates SREBP2 specifically through the SCAP SSD, we focused on the evolutionarily conserved residue Tyr298 (Y298), which has been functionally characterized in yeast SCAP SSD [39]. In yeast systems, Y298 localizes to an allosteric network hub within the SSD and participates in SCAP‐INSIG1/2 complex assembly. Mutations at this site confer sterol insensitivity and lead to constitutive SREBP activation [39]. In contrast, mutating D428 to alanine (D428A) locks SCAP in an INSIG‐bound conformation, even under sterol‐depleted conditions that would otherwise promote their dissociation. Unable to release from INSIG, SCAP fails to deliver SREBPs to the Golgi for processing, resulting in sustained suppression of the SREBP pathway. Similarly, the Q432A mutant forms a stable complex with INSIG without requiring 25‐HC (a cholesterol signal mimic), maintaining the bound state even under sterol‐deficient conditions [31]. This opposing regulatory function provides pivotal evidence supporting pharmacological targeting of the SSD by PD. We hypothesized that Y298 mutation may impair SCAP membrane localization or proteolytic cleavage sensitivity, thereby weakening PD's ability to activate cholesterol synthesis. However, the role of these sites in human SCAP and the mechanism of PD targeting remain unclear.

Sequence alignment across species confirmed high conservation of Y298, D428, and Q432 within the SSD (Figure S4E). We constructed SCAP mutants, including SSD deletion and point mutations (Y298C, D428A, Q432A) to examine their roles in PD response (Figure 4F). ‌Co‐IP in SCAP KD GC cells (Figure S4F) showed that SSD deletion markedly reduced SCAP‐INSIG1 binding while enhancing SCAP‐SREBP2 interaction (Figure 4G), consistent with the cholesterol accumulation phenotype observed previously (Figure 2J). Importantly, ‌PD treatment did not further strengthen SCAP‐SREBP2 binding in the SSD‐deletion mutant, indicating that an intact SSD is required for PD‐induced complex formation (Figure 4G). The Y298C mutation also weakened SCAP‐INSIG1 association and enhanced SCAP‐SREBP2 interaction; however, this enhanced binding was not further increased by PD (Figure 4H). In line with constitutive activation, the Y298C mutation elevated nuclear SREBP2 and cholesterol levels (Figure 4I,J,L; Figure S4H), identifying Y298 as a critical residue for PD‐mediated activation of cholesterol biosynthesis via SCAP SSD.

We next examined whether the conserved residues Asp428 (D428) and Gln432 (Q432) are involved in PD‐induced SREBP2 activation. Conversely, the D428A mutation strengthened SCAP‐INSIG1 binding and reduced SCAP‐SREBP2 interaction (Figure 4K). However, in D428A‐expressing cells, PD treatment not only partially restored SCAP‐SREBP2 binding but also increased cholesterol levels (Figure 4K,L; Figure S4H), indicating that this mutation cannot fully abolish PD‐mediated activation. Similarly, the Q432A mutation enhanced SCAP‐INSIG1 association and diminished SCAP‐SREBP2 binding, yet PD partially rescued SCAP‐SREBP2 interaction and cholesterol levels in Q432A‐expressing cells (Figure S4G,H; Figure 4L). These findings indicate that, although D428 and Q432 are evolutionarily conserved and their mutants lock SCAP in an INSIG‐bound state, the partial response of mutant cells to PD suggests that PD can still partially restore pathway activity through other interactions within the SSD, pointing to the existence of multiple regulatory nodes within this domain.

### 2.7 Cholesterol Overload‐Driven ER Stress Triggers Nrf1 Nuclear Translocation and Impairs Cholesterol Efflux

**FIGURE 5:**
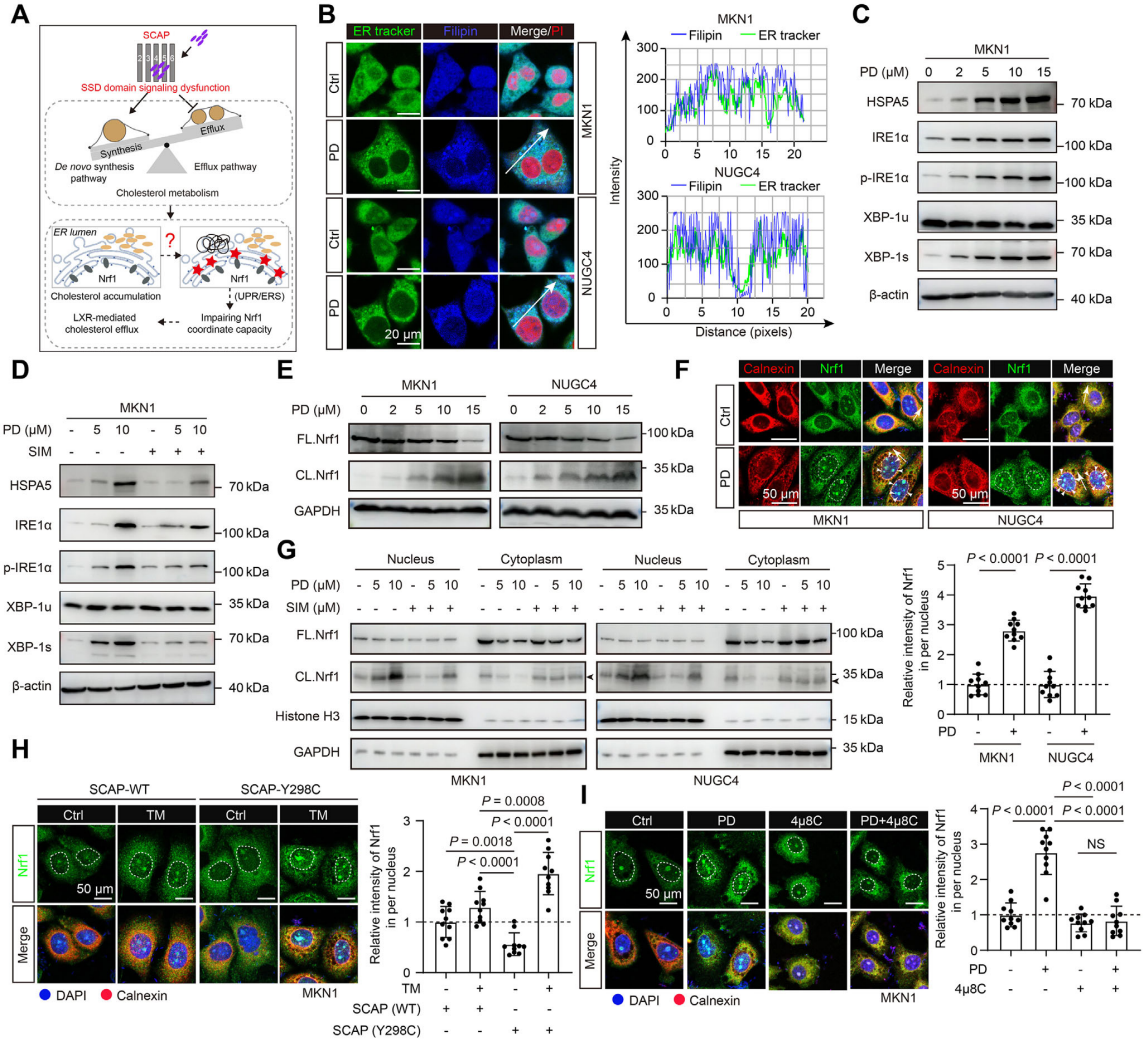
PD mediates the cholesterol‐dependent ER stress to force nuclear translocation of Nrf1. (A) Proposed model: pathological cholesterol accumulation induces ER stress/UPR, impairing the function of ER‐anchored Nrf1 and its coordination of cholesterol efflux. ER, endoplasmic reticulum; UPS, unfolded protein response. (B) Quantification of cholesterol (Filipin) and ER‐Tracker colocalization. Colocalization analysis was performed in regions indicated by white arrows using ImageJ. Scale bar = 20 µm. (C, D) Immunoblots of IRE1α/XBP‐1 pathway proteins in MKN1 cells treated with vehicle, PD alone (C), or PD combined with simvastatin (SIM) (D). (E) Immunoblots of Nrf1 in MKN1 and NUGC4 cells treated with vehicle or indicated PD concentrations. FL.Nrf1, full‐length Nrf1 protein; CL.Nrf1, cleaved Nrf1 protein. (F) Representative immunofluorescence images showing Nrf1 colocalization with the ER (marked by calnexin). Bar graph quantifies nuclear Nrf1 intensity. Colocalization analysis performed in regions marked by white arrows (see Figure S5C for additional information). Scale bar = 50 µm. (G) Nuclear and cytoplasmic Nrf1 levels in MKN1 and NUGC4 cells treated with vehicle, PD alone, or PD combined with SIM. (H) Nrf1‐ER colocalization in SCAP‐knockdown MKN1 cells expressing Flag‐SCAP WT or Y298C mutant, treated with vehicle or tunicamycin (TM) for 24 h. Graph shows nuclear Nrf1 intensity. White dashed circle indicates nuclei. Scale bar = 50 µm. (I) Nrf1‐ER colocalization in MKN1 cells treated with PD, 4µ8C (IRE1α inhibitor), or both; with nuclear Nrf1 intensity quantified in the bar graph. White dashed circle indicates nuclei. Scale bar = 50 µm. NS, not significant.

Cellular cholesterol‐loading capacity is intrinsically limited, with the ER maintaining <5% of total cellular sterols, rendering it highly susceptible to overload [40, 41]. Excess cholesterol influx compromises ER membrane fluidity and impairs the function of resident enzymes, ultimately triggering dysregulated adaptive responses [42]. Our findings indicate that PD targeting of the SCAP SSD suppresses the LXR‐driven cholesterol efflux machinery (Figure 3K; Figure S3I), suggesting that upstream regulatory factors linking this effect may involve ER cholesterol accumulation. Under cholesterol stress, the LXR‐mediated efflux pathway plays a key role in alleviating sterol burden. Nuclear factor erythroid 2‐related factor 1 (Nrf1/NFE2L1), an ER‐transmembrane sterol sensor, helps maintain cholesterol homeostasis by transcriptionally repressing CD36‐mediated inflammatory cascades and derepressing LXR activity [29]. We therefore hypothesized that PD‐induced ER cholesterol accumulation disrupts Nrf1's coordinate function, impairing its ability to coordinate compensatory pathways required for cholesterol homeostasis under metabolic stress (Figure 5A). Strong colocalization of Filipin and ER‐tracker confirmed substantial cholesterol deposition in the ER following PD treatment (Figure 5B). Western blot analysis showed dose‐dependent activation of the IRE1α/XBP‐1 ER stress pathway upon PD exposure (Figure 5C; Figure S5A), which was effectively counteracted by SIM co‐treatment (Figure 5D; Figure S5B), confirming that PD induces cholesterol‐dependent ER stress.

We next examined the effects of PD on Nrf1 expression and subcellular localization. Western blot analysis revealed decreased full‐length Nrf1 (FL.Nrf1) levels but increased cleaved Nrf1 (CL.Nrf1) levels after PD treatment (Figure 5E). Functionally, following deglycosylation at the ER membrane, Nrf1 undergoes p97/VCP complex‐mediated retrotranslocation of its transactivation domain from the ER lumen to the cytosolic compartment, where it is cleaved to release a transcriptionally active nuclear isoform [29]. As expected, IF experiments utilizing Calnexin as a specific ER marker revealed substantial nuclear accumulation of Nrf1 upon PD treatment (Figure 5F), and colocalization analysis confirmed reduced ER retention of Nrf1 (Figure S5C). Nuclear‐cytoplasmic fractionation assays further validated that PD significantly increased CL.Nrf1 levels in the nucleus (Figure 5G). Importantly, SIM co‐treatment markedly reversed PD‐induced Nrf1 nuclear translocation (Figure 5G), supporting the role of ER cholesterol overload as the primary driver of this process.

To determine whether cholesterol‐dependent ER stress triggers Nrf1 nuclear translocation, we treated wild‐type and SCAP‐Y298C mutant cells with the ER stress inducer tunicamycin (TM). In DMSO‐treated SCAP‐Y298C cells, Nrf1 remained cytoplasmic; however, TM induced robust nuclear accumulation, an effect less pronounced in wild‐type cells (Figure 5H), suggesting that ER stress alone is insufficient to trigger translocation in the absence of cholesterol dysregulation. Notably, co‐treatment with the ER stress inhibitor 4µ8C significantly reversed PD‐induced Nrf1 nuclear translocation (Figure 5I). These results demonstrate that cholesterol overload‐dependent ER stress is both necessary and sufficient for Nrf1 nuclear translocation, and that transcriptional repression of LXRs by nuclear Nrf1 may mechanistically explain the impairment of cholesterol efflux following PD treatment.

### 2.8 PD Induces ER Stress‐Dependent Ferroptosis in GC

**FIGURE 6:**
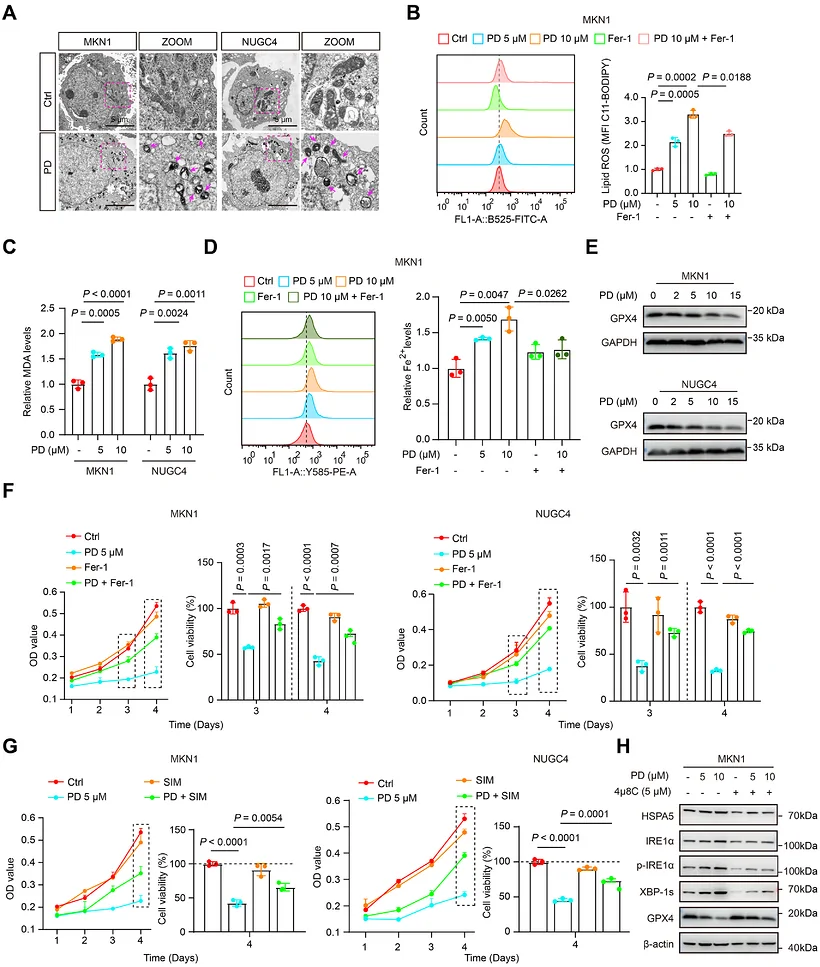
PD induces ER stress‐dependent ferroptosis via ER cholesterol accumulation in GC. (A) Representative transmission electron microscopy images showing mitochondrial morphology in MKN1 and NUGC4 cells after PD treatment. Pink arrows indicate mitochondrial shrinkage and cristae loss. Scale bar = 5 µm. (B) Representative flow cytometry plots and quantification of lipid ROS levels in MKN1 cells treated with vehicle, PD alone, or PD combined with 5 µm ferrostatin‐1 (Fer‐1) (*n* = 3 biological replicates). (C) Relative MDA levels in MKN1 and NUGC4 cells after 24‐hour PD treatment. (D) Representative flow cytometry plots and quantification of the Fe^2+^ levels in MKN1 cells treated with vehicle, PD alone, or PD combined with 5 µm Fer‐1 (*n* = 3 biological replicates). (E) Immunoblots of GPX4 expression in MKN1 and NUGC4 cells treated with vehicle or indicated PD concentrations for 24 h. (F, G) Proliferation of MKN1 and NUGC4 cells treated with vehicle, PD alone, or PD combined with 2 µm Fer‐1 (F) or 1 µm simvastatin (SIM) (G) for the indicated time (*n* = 3 biological replicates). (H) Immunoblots of IRE1α/XBP‐1 pathway activation and GPX4 expression in MKN1 cells treated with vehicle, PD alone, or PD combined with the IRE1α inhibitor 4µ8C. NS, not significant.

The role of SSD‐mediated cholesterol accumulation in ER stress and subsequent cell fate regulation remains unclear. Our proteomic analysis showed significant enrichment of ferroptosis‐related pathways in PD‐treated cells compared with vehicle control (Figure 3G; Figure S3F), suggesting a potential mechanistic link. Morphological and ultrastructural analyses revealed that PD induced marked mitochondrial abnormalities, including shrinkage, cristae dissolution, and outer membrane fragmentation‐all established hallmarks of ferroptosis (Figure 6A). Consistent with these findings, flow cytometry using C11‐BODIPY staining demonstrated a dose‐dependent increase in lipid reactive oxygen species (lipid ROS) in PD‐treated cells, which was markedly attenuated by the ferroptosis inhibitor Ferrostatin‐1 (Fer‐1) (Figure 6B; Figure S6A). Malondialdehyde (MDA) assays further confirmed elevated lipid peroxidation in a dose‐dependent manner (Figure 6C). Moreover, ferrous iron assays indicated a PD‐induced, dose‐dependent rise in Fe^2^^+^ that was partially reversed by Fer‐1 (Figure 6D; Figure S6B). Notably, PD also reduced protein levels of the key ferroptosis suppressor GPX4 in a dose‐dependent manner (Figure 6E). Co‐treatment with Fer‐1 and PD confirmed that Fer‐1 rescues PD‐induced cell death (Figure 6F), collectively demonstrating that pharmacological targeting of the SCAP SSD triggers ferroptosis in cancer cells.

To examine whether cell survival is affected by cholesterol metabolism dysregulation and subsequent ER stress cascades, we combined SIM with PD. SIM treatment effectively reversed PD‐induced suppression of viability (Figure 6G). Furthermore, co‐treatment with the IRE1α inhibitor 4µ8C and PD suppressed XBP‐1s and restored GPX4 expression (Figure 6H; Figure S6C), indicating that SCAP SSD inhibition‐triggered ferroptosis depends on ER stress activation driven by pathological cholesterol accumulation.

As summarized in Figure S6D, PD targets the SCAP SSD, disrupts SCAP‐INSIG1 interaction, and promotes SCAP‐SREBP2 complex trafficking and maturation, thereby activating de novo cholesterol synthesis. Given the ER's central role in cholesterol synthesis, we investigated the downstream consequences of PD‐induced cholesterol accumulation. Nrf1, an ER membrane‐anchored cholesterol sensor, showed enhanced nuclear translocation upon cholesterol overload‐dependent ER stress, confirming that persistent ER cholesterol accumulation drives Nrf1 activation. In PD‐treated tumor cells, downregulation of cholesterol efflux transporters (e.g., *ABCA1* and *ABCG1*) was consistent with Nrf1‐mediated suppression of LXRs, revealing PD's dual role in promoting cholesterol synthesis and inhibiting efflux. Within this framework, we have elucidated the connection between ER cholesterol overload and cell fate, establishing ER stress‐dependent ferroptosis as the principal mechanism of PD‐mediated tumor suppression.

### 2.9 PD Suppresses GC Tumor Growth via ER Stress‐Dependent Ferroptosis In Vivo

**FIGURE 7:**
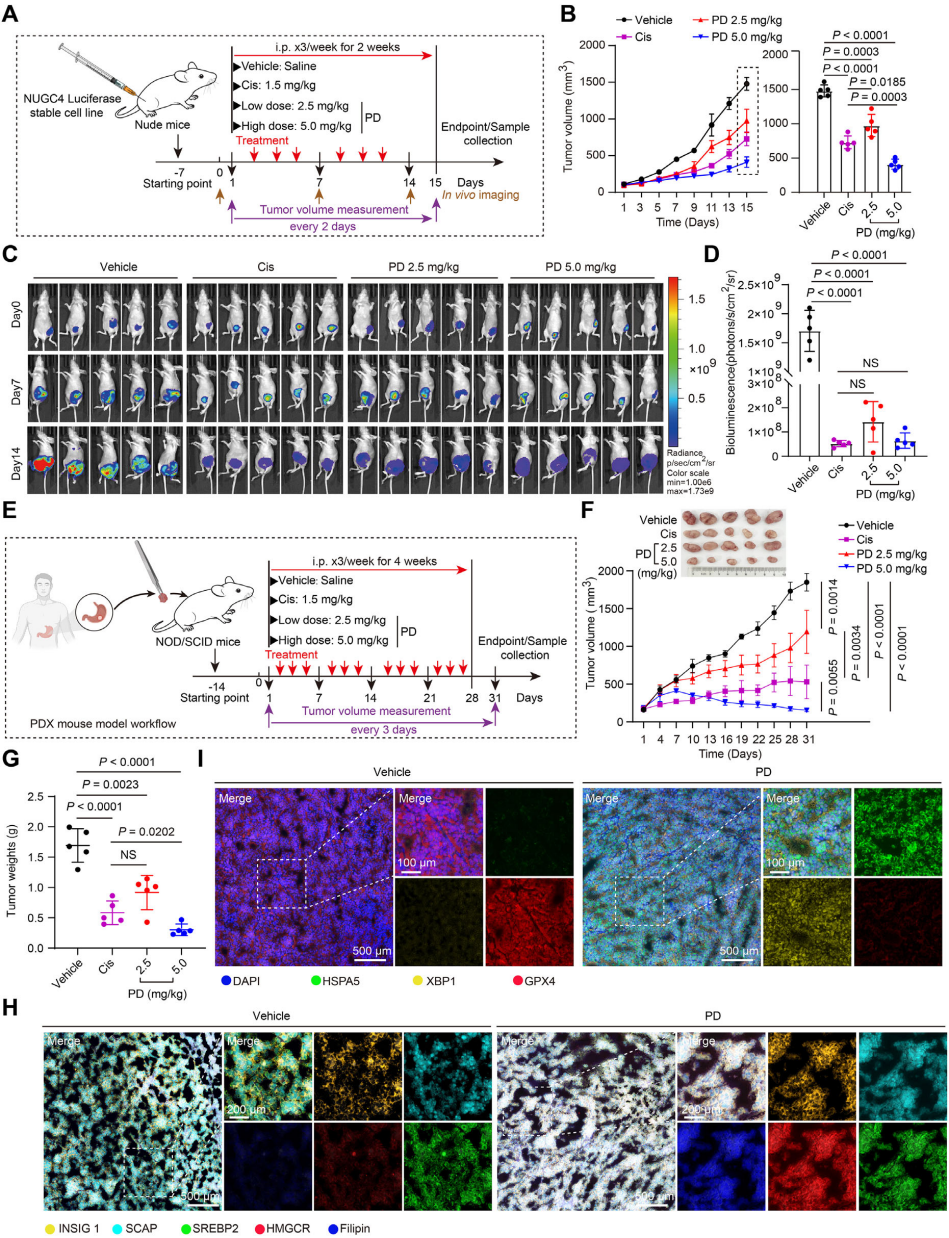
PD suppresses GC tumor growth by remodeling cholesterol metabolism in vivo. (A) Experimental setup for evaluating PD in a cell line‐derived xenograft (CDX) model. NUGC4‐luciferase cells were injected subcutaneously into nude mice. After 7 d, mice received intraperitoneal (i.p.) injection of vehicle, PD (2.5 or 5.0 mg kg^−1^), or cisplatin (Cis, 1.5 mg kg^−1^) three times per week for 2 weeks. (B) Growth curves of NUGC4 CDX tumors. (C) In vivo bioluminescence imaging of mice from each treatment group (*n*  =  5 mice per group). (D) Quantification of bioluminescence intensity from in vivo imaging. (E) Experimental setup for evaluating PD in a patient‐derived xenograft (PDX) model. NOD/SCID mice bearing PDX tumors received i.p. injections of vehicle, PD (2.5 or 5.0 mg kg^−1^), or Cis (1.5 mg kg^−1^) three times per week for 4 weeks. (F, G) Tumor growth curves (F) and final tumor weights (G) of PDX tumors (*n*  =  5 mice per group). (H) Representative multiplex immunohistochemical image showing SCAP, SREBP2, HMGCR, INSIG1, and Filipin staining in PDX tumor tissues. Scale bar = 500 µm. (I) Representative multiplex immunohistochemical image showing HSPA5, XBP‐1, and GPX4 in PDX tumor tissues. Scale bar = 500 µm. NS, not significant.

To evaluate the therapeutic potential of SCAP SSD targeting in GC, we established a cell line‐derived xenograft (CDX) model. Mice were randomized into four groups receiving physiological saline, cisplatin (Cis), or two doses of PD (Figure 7A). PD treatment resulted in significant, dose‐dependent suppression of tumor growth compared with the vehicle group (*p* < 0.001) (Figure 7B–D).

To further validate these findings, we developed a patient‐derived xenograft (PDX) model using tumor tissue from a treatment‐naïve primary GC patient. Fifteen days after implantation, mice were randomized into treatment groups and administrated saline, Cis, or PD for four weeks, with tumor volume and weight assessed as endpoints (Figure 7E). PD again exhibited robust, dose‐dependent inhibition of tumor growth, with the high dose showing optimal efficacy while preserving normal physiological status. This effect was superior to that of Cis, which induced significant body weight loss and renal damage (Figure 7F,G; Figure S7A–C). Together, these data underscore the therapeutic promise of SCAP SSD targeting in GC.

We next performed molecular profiling of tumor tissues from both models. Cholesterol staining of freshly harvested samples showed that PD increased expression of key cholesterol biosynthesis regulatory proteins (SREBP2 and HMGCR) and elevated total cholesterol levels without altering SCAP expression (Figure 7H; Figure S7D). mIHC revealed that PD elevated ER stress markers (XBP‐1 and HSPA5), while suppressing GPX4, relative to controls (Figure 7I; Figure S7E), consistent with our in vitro findings (Figure 5C; Figure 6E). Collectively, these in vivo results support the conclusion that PD‐mediated SCAP SSD targeting suppresses tumor growth through cholesterol metabolism‐induced ER stress and subsequent ferroptosis.

### 2.10 PD Inhibits Patient‐Derived Organoid Growth and Spheroid Formation by Disrupting Cholesterol Metabolism and Inducing Ferroptosis

**FIGURE 8:**
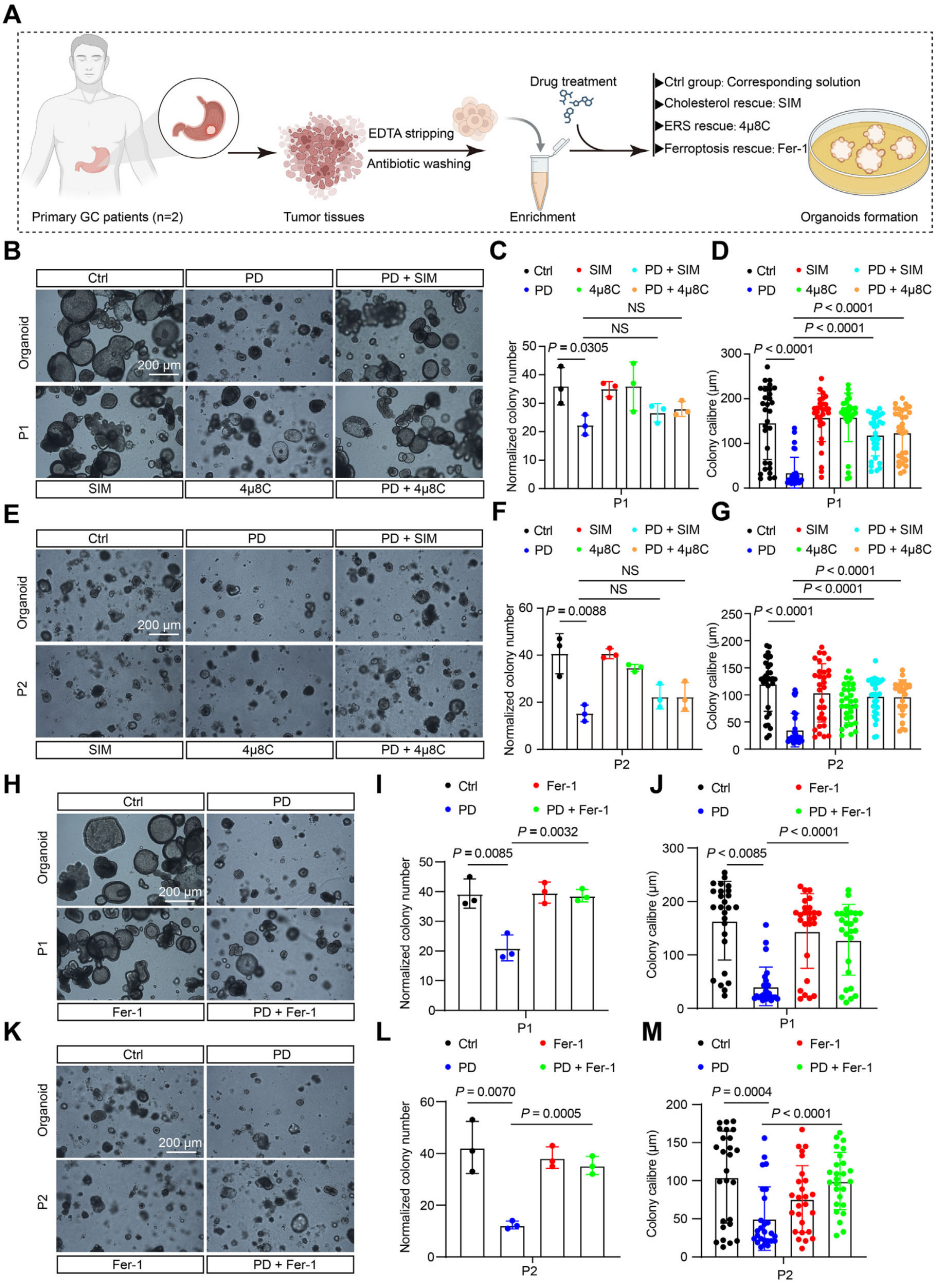
PD inhibits GC tumor growth through ER stress‐dependent ferroptosis in patient‐derived organoid model. (A) Experimental workflow for evaluating PD in patient‐derived organoids (PDOs) from two treatment‐naïve GC patients. PDOs were first assessed in vitro to minimize confounding factors, followed by pharmacological intervention with vehicle, PD alone (5 µm), or PD combined with 2 µm 4µ8C or 0.5 µm SIM or 2 µm Fer‐1. (B–M) Representative bright‐field images (B, E, H, K) of PDOs after treatment with indicated compounds. Quantification of organoid counts (C, F, I,L) and diameters (D, G, G,M) is shown for each. P1, Patient 1. P2, Patient 2. Scale bar = 200 µm. NS, not significant.

To evaluate the clinical translational potential of SCAP SSD targeting in GC, we performed drug testing using patient‐derived organoids (PDOs) from two treatment‐naïve GC patients (P1 and P2) to minimize potential confounding factors (Figure 8A). PD treatment significantly suppressed organoid growth and spheroid formation, as shown by marked reductions in both spheroid number and diameter (Figure 8B–G). Notably, although SIM or 4µ8C did not fully restore spheroid counts, both agents partially reversed the reduction in spheroid diameter (Figure 8B–G). These results highlight the essential contribution of ER cholesterol accumulation and subsequent ER stress to the anti‐tumor effects of SCAP SSD targeting, supporting the disruption of cholesterol homeostasis as a viable therapeutic strategy in GC.

We further asked whether PD's inhibitory effects in PDOs depend mechanistically on ferroptosis. Co‐treatment with Fer‐1 significantly reversed rescued organoid growth and spheroid formation suppressed by PD (Figure 8H–M), confirming that ferroptosis is the primary mode of PD‐mediated cell death in these clinically relevant models.

## 3 Discussion

Tumor cells exhibit a distinct vulnerability to cholesterol dyshomeostasis, as metabolic reprogramming in the tumor microenvironment hijacks sterol flux to support oncogenesis. Cholesterol‐derived metabolites promote cancer progression and suppress immune surveillance. Accumulating preclinical and clinical evidence indicates that strategic modulation of cholesterol metabolism can effectively inhibit tumor growth and remodel antitumor immunity [43], highlighting the therapeutic potential of targeting cholesterol homeostasis. However, the lack of precise molecular targets and effective agents has hindered clinical validation of this approach.

SCAP is a central cholesterol sensor that functions as a molecular switch, regulating SREBP activity and nuclear translocation through conformational changes, thereby linking lipid metabolism to cellular function. Its domain architecture (L1–L7) reveals druggable conformational switches that represent potential targets for metabolic and immunomodulatory intervention [16, 44, 45]. In our GC cohort, multi‐omics analyses revealed tumor‐specific SCAP overexpression strongly associated with poor prognosis. Pathological specimens consistently showed elevated SCAP expression in tumors compared with matched adjacent tissues, along with coordinated upregulation of de novo cholesterol synthesis proteins and increased cholesterol content in malignant tissues, suggesting that tumor cells undergo metabolic reprogramming to meet the biosynthetic demands. These findings provide mechanistic insights into how cholesterol metabolism becomes specialized during tumor evolution, establishing a rationale for targeting SCAP therapeutically. Given its critical role in GC, we hypothesized that pharmacological targeting of the SCAP SSD could disrupt cholesterol homeostasis for therapeutic benefit. Clinical validation supported this hypothesis and revealed a key knowledge gap: although existing SCAP inhibitors modulate protein stability or downstream signaling [46], none directly target the SSD.

Using the SSD as a druggable pocket, we performed structure‐based high‐throughput screening and identified compounds capable of directly engaging the SSD and disrupting cellular sterol sensing, while sustaining SREBP2 activation, a hallmark of bypassed homeostatic feedback. PD and DGT were validated as SSD‐targeted inhibitors, with cryo‐EM structural analysis of the SCAP‐INSIG2 complex confirming DGT binding within the SSD pocket [30], authenticating our screening approach. PD‐induced metabolic dysregulation occurred through three interconnected mechanisms: first, it disrupted cellular sterol sensing, permitting sustained de novo synthesis under cholesterol‐saturated conditions. Second, it downregulated cholesterol efflux transporters (ABCA1, ABCG1), impairing cholesterol elimination; and third, it led to pathological cholesterol accumulation in the ER, indicating systemic failure. Notably, although SCAP protein levels remained unchanged, PD promoted SREBP2 maturation and transcriptional activation of biosynthetic genes.

These findings align with the roles of key SSD residues (Y298, L315, and D443), which are essential for sterol sensing [30, 47, 48, 49, 50]. Mutations such as hamster SCAP Y298C, L315F, and D443N disrupt SCAP‐INSIG interaction and cause constitutive SREBP2 cleavage and activation independent of sterol levels. Conversely, mutations that impair SCAP‐INSIG dissociation (e.g., D428A, Q432A) abolish SREBP2 proteolytic processing even under sterol‐depleted conditions [31, 51, 52]. Among the well‐characterized loss‐ or gain‐of‐function (LOF or GOF) SCAP mutations, our data indicate that PD targets Y298 residue within the SSD to conformationally stabilize the SREBP2‐engaged state, thereby sustaining de novo cholesterol biosynthesis.

Metabolic adaptations preserve cellular homeostasis; however, chronic nutrient excess can lead to pathological metabolite accumulation, overwhelming homeostatic defenses and driving metabolic disorder [53]. The ER serves as a central adaptive organelle that maintains proteostatic and lipid metabolic balance [54, 55, 56]. In exploring the mechanism of cholesterol metabolic dysfunction, we focused on cellular cholesterol efflux machinery. Our findings indicate that ER cholesterol accumulation is both a cause and consequence of efflux impairment. PD‐induced cholesterol accumulation and concurrent efflux deficiency reflect disrupted ER sensing, mediated by the ER‐resident cholesterol sentinel Nrf1. Under adaptive conditions, membrane‐tethered Nrf1 monitors luminal cholesterol fluctuations and coordinates CD36/LXR regulatory networks [29]. We found that PD increased the proteolytic fragment of Nrf1, promoted its nuclear translocation, and suppressed LXR transcriptional activity, indicating cholesterol‐induced sensing failure via two mechanisms: Nrf1 mislocalization disrupting sterol surveillance, and ER membrane alterations due to sterol saturation. Our study establishes that cholesterol overload‐dependent ER stress is sufficient to trigger Nrf1 nuclear translocation, positioning Nrf1 as a critical switch translating cholesterol overload into transcriptional repression. Further studies are needed to fully elucidate the cholesterol‐Nrf1‐LXR axis.

Cellular homeostasis requires integration of nutrient management with adaptive systems that counteract biochemical damage [29, 53]. We demonstrate that PD‐induced ER cholesterol accumulation determines cell fate via ferroptosis, as shown by ultrastructural alterations and elevated lipid ROS. This process results from pathological cholesterol saturation exceeding organelle buffering capacity, leading to proteotoxic stress that overwhelms adaptive responses [56, 57, 58, 59]. A key piece of evidence supporting this conclusion is that PD‑mediated ferroptosis can be partially reversed by cholesterol clearance. Although Nrf1 has been implicated in the pathological accumulation of cholesterol, recent studies have shown that Nrf1 can also promote ferroptosis resistance by maintaining GPX4 expression in an Nrf2‑independent manner [60], highlighting a close functional link between Nrf1 and ferroptosis regulation. As a core regulator of antioxidant responses, Nrf2 plays a critical role in suppressing ferroptosis [61]. Beyond its involvement in cholesterol accumulation, PD‑mediated changes in the nucleocytoplasmic distribution of Nrf1 may influence Nrf1 signaling output and potentially intersect with ferroptosis via pathway crosstalk. However, the underlying mechanisms remain to be further investigated. In summary, targeting cholesterol metabolism represents a feasible anti‐tumor strategy, and studying SCAP's role in cholesterol sensing and metabolic balance offers a clinically valuable direction. Our findings provide new insights for therapeutic target identification and innovative GC treatment development.

## 4 Materials and Methods

### 4.1 Mice

Four‐ to five‐week‐old female BALB/c‐nude mice and NOD/SCID mice were purchased from Hangzhou Qizhen Laboratory Animal Technology Co., Ltd. The mice were raised in the standard specific pathogen‐free (SPF)‐grade animal house of the Hangzhou Institute of Medicine (HIM), Chinese Academy of Sciences. The housing conditions included a 12 h light/12 h dark cycle at 20–22°C and 40–70% humidity. In accordance with the protocols for experimentation on animals (National Institutes of Health Publication No. 85‐23, revised 1996) and approved by the Hangzhou Institute of Medicine, Chinese Academy of Sciences (Approval No. AP2025‐12‐0670). For all xenograft studies, animals were randomly allocated to experimental groups. To maintain blinding, cell injections and tumor transplantations were performed independently by one team of researchers, while another team carried out subsequent treatments and tumor measurements.

### 4.2 Collections and Preparation of Clinical Specimens

This study utilized samples from 75 patients at the Zhejiang Cancer Hospital from April 2009 to April 2018. The Research Ethics Committees of Zhejiang Cancer Hospital approved the study (No. IRB‐2021‐288). These samples include paired normal and tumor tissues; see our previous study for additional information [62]. The fresh tissues from 15 GC patients used for analysis of SCAP expression characteristics and the primary GC tissue used for the patient‐derived xenograft model in this study were all obtained from the Pathology Department of Zhejiang Cancer Hospital. A portion of the pathological tissues underwent frozen sectioning by the pathology department (primarily for Filipin staining), while another portion was fixed, dehydrated, paraffin‐embedded, sectioned, and subsequently subjected to multiplex immunohistochemical (mIHC) analysis.

### 4.3 Multiplex Immunohistochemical Analysis

The mIHC assay was performed on FFPE GC tissues (2 µm) using a tyramine signal amplification (TSA)‐based workflow. Following deparaffinization (xylene/ethanol), antigen retrieval (10 mM citrate buffer, 95°C), and blocking (H_2_O_2_/non‐specific), sequential labeling of SCAP antibody (Proteintech, 12266‐1‐AP), SREBP2 antibody (Invitrogen, PA5‐24167), HMGCR antibody (Santa Cruz, sc‐271595), and INSIG1 antibody (Santa Cruz, sc‐390504) was achieved using horseradish peroxidase (HRP)‐conjugated secondary antibodies coupled to tyramide‐coupled Opal fluorophores (480‐690 nm). Multispectral imaging (Akoya Vectra Polaris) coupled with InForm software enabled spectral unmixing, epithelial/stromal compartmentalization, and single‐cell phenotyping.

### 4.4 Subcutaneous Xenograft and Patient‐Derived Xenografts (PDX) Models

Female BALB/c nude mice (5 weeks old) were used for in vivo studies. NUGC4‐luc cells (5 × 10^5^ cells in 100 µL PBS) were subcutaneously implanted into the left flank to establish a xenograft model. Mice were randomized (*n* = 5 mice per group) into four groups 7 d after implantation: Vehicle control: 200 µL sterile saline (i.p.); PD (2.5 or 5.0 mg kg^−1^ body weight in saline, i.p.); cisplatin (Cis, 1.5 mg kg^−1^ body weight in saline, i.p.). Treatments were administered every 2 d for a total of seven injections. Tumor progression was quantified weekly by bioluminescence imaging (IVIS Spectrum CT, PerkinElmer) following intraperitoneal injection of D‐luciferin (Yeasen, 40902ES01). Body weight was recorded every two days to assess systemic toxicity.

PDX models were established by the Zhejiang Cancer Hospital (Hangzhou, China). Female NOD/SCID mice (4 weeks old) were housed under SPF conditions with autoclaved feed and acidified water. After one week of acclimatization, PDX tumor fragments (2×2×2 mm) were subcutaneously implanted into the left flank. Mice were randomly divided into four groups (*n* = 5 mice per group) when tumors reached 50 mm^3^ (volume = length × width^2^/2). The vehicle control received 200 µL saline, while the positive control group was treated with 200 µL of Cis (1.5 mg kg^−1^ body weight in saline). PD‐treated groups were administrated 200 µL of PD at 2.5 mg kg^−1^ and 5.0 mg kg^−1^ body weight, respectively, and tumor volume and body weight were measured three times a week for 4 weeks using a digital caliper and a precision balance, respectively. Relative tumor volume and tumor weight were calculated to assess drug efficacy. Body weight was recorded every three days to assess systemic toxicity. Terminal tumors and major organs (heart, liver, spleen, lung, and kidney) were fixed in 4% PFA for IHC and H&E histopathological analysis.

### 4.5 Protein Extraction and Immunoblotting Analysis

The general process is similar to that used in our previous research [63, 64]. Cell lysates were prepared using ice‐cold RIPA lysis buffer supplemented with 1×protease inhibitor cocktail (1:100 dilution of protease inhibitor stock solution) (Biosharp, BL1426A). Total protein was quantified using the BCA protein assay kit (Beyotime Biotechnology, P0011) with bovine serum albumin as the standard. Equal aliquots of protein were resolved on 10% Tris‐glycine SDS‐polyacrylamide gels under denaturing conditions, with β‐actin serving as an internal normalization control. Electrophoretically separated proteins were transferred to 0.22 µm PVDF membranes (Roche, 03010040001) using a transfer system (Bio‐Rad). Membranes were blocked with 5% non‐fat dry milk in TBST for 2 h at RT. Immunoblotting was performed using the following validated antibodies: anti‐SCAP (1:1000; Abcam, ab125186), anti‐INSIG1 (1:500; Santa Cruz, sc‐390504), anti‐SREBP2 (1:500; Santa Cruz, sc‐13552), anti‐HMGCR (1:500; Santa Cruz, sc‐271595), anti‐GPX4 (1:1000; Cell Signaling Technology, 52455S), anti‐IRE1α(1:1000; Cell Signaling Technology, 3294S), anti‐p‐IRE1α(1:1000; Abcam, ab48187), anti‐XBP‐1(1:1000; Cell Signaling Technology, 12782S), anti‐HSPA5 (1:1000; Cell Signaling Technology, 3177S), and anti‐β‐actin (1:1000; Beyotime Biotechnology, AF0003) (refer to Table S2 for more instructions on using the antibody). All primary incubations were performed overnight at 4°C with gentle shaking. After three 5 min TBST washes, the membranes were probed with horseradish peroxidase (HRP)‐conjugated secondary antibodies (anti‐rabbit IgG, 1:5000; Cell Signaling Technology, 7074S) or (anti‐mouse IgG, 1:5000; Cell Signaling Technology, 7076S) for 2 h at RT. Chemiluminescence signals were developed using ECL substrate (Biosharp, BL520B) and captured using an Amersham ImageQuant 800 system (Cytiva). Quantification of band intensities was performed using ImageJ v1.51k with background subtraction.

### 4.6 Co‐Immunoprecipitation Assay (Co‐IP)

Detailed experimental methods have been previously described [65]. Cells were harvested after experimental treatments and washed twice with ice‐cold PBS. Pelleted cells were lysed in 600 µL IP buffer containing 1 × protease inhibitor cocktail (Biosharp, BL1426A) for 2 h at 4°C on a rotary shaker. Lysates were clarified by centrifugation at 3500 × rpm for 5 min at 4°C. Subsequently, 2.5 µL of mouse IgG (Beyotime Biotechnology, A7028) or rabbit IgG (Beyotime Biotechnology, A7016) was incubated with the lysates for 4 h at 4°C, followed by the addition of 25 µL of Protein A+G agarose (Beyotime Biotechnology, P2055) for 3 h at 4°C. After removal of the beads (3500 × rpm, 5 min, 4°C), the supernatants were immunoprecipitated with specific primary antibodies overnight at 4°C. Immune complexes were captured with 35 µL Protein A+G agarose during 4 h incubation with rotation. The bead‐bound complexes were washed five times with ice‐cold PBS, and 50 µL of 1 × loading buffer (Beyotime Biotechnology, P0015) was added to the antigens and boiled at 100°C for 10 min.

### 4.7 Cellular Thermal Shift Assay (CETSA)

MKN1, NUGC4, and HEK293T cells were treated with PD (20 µm) or vehicle control (0.1% DMSO) for 2 h at 37°C under 5% CO_2_, or with PD (0.0001–100 µm) for 24 h. Following treatment, cells were harvested, washed twice with ice‐cold PBS, and lysed in ice‐cold PBS buffer containing 1 × protease inhibitor cocktail (Biosharp, BL1426A). Lysates were aliquoted into PCR tubes and subjected to thermal gradients (41°C, 44°C, 47°C, 50°C, 53°C, 56°C, and 59°C) for 3 min or concentration gradients for 37°C and 50°C using a C1000 Touch Thermal Cycler (Bio‐Rad cat #1851197), followed by immediate cooling on liquid nitrogen for 5 min, thaw at room temperature. After three cycles, 5× protein loading buffer was added to the final samples for Western blot analysis.

### 4.8 Measurement of Total Cholesterol

Total cholesterol was assayed using the Amplex Red Cholesterol Test Kit (Beyotime, S0211S) following the manufacturer's protocol. Briefly, PD‐treated MKN1 and NUGC4 cell samples (1 × 10^6^ cells) were collected. Cells were lysed by the addition of 200 µL of BeyoLysis Buffer A. The cells were subsequently centrifuged at 12 000 × *g* for 5 min at 4°C, after which the supernatant was removed for subsequent assays. Cholesterol assay buffer, Amplex Red, cholesterol esterase and enzyme mixture were added to the supernatant, and the mixture was allowed to react for 30 min at 37°C under light protection. The absorbance of each well at 570 nm was measured using a microplate reader (Spark TECAN, Switzerland).

### 4.9 Filipin III Staining

MKN1 and NUGC4 cells were seeded at a density of 6 × 10^3^ and 4 × 10^3^ cells per well, respectively, in 24‐well chambered cover glass systems (Biosharp, BS‐15‐RC) and cultured for 24 h under standard conditions (37°C, 5% CO_2_). Cells were subjected to the indicated treatment and then fixed with 4% PFA followed by incubation with 50 µg mL^−1^ Filipin III (MCE, HY‐N6718) staining solution at RT and away from light for 0.5 h, after PD and U‐18666A treatment. Furthermore, the nuclei were stained with PI stain. Fluorescence images of filipin III staining were taken via a Nikon A1R HD25 confocal microscope. The filipin III staining quantification was analyzed by ImageJ.

To spatially resolve cholesterol accumulation in GC patients, we employed fluorescence‐based filipin III histochemistry. Briefly, fresh‐frozen OCT‐embedded tissues were sectioned (10 µm, Thermo Scientific Cryostar NX70), fixed in 10% neutral buffered formalin, washed thrice in PBS, stained with filipin III (50 µg mL^−1^, Sigma) for 20 min, and counterstained with PI. After a final rinse in PBS, samples were immediately examined under an immunofluorescence microscope (Nikon, A1R HD25).

### 4.10 Immunofluorescence (IF) Assay

MKN1 and NUGC4 cells were seeded at a density of 6 × 10^3^ and 4 × 10^3^ cells per well, respectively, in 24‐well chambered cover glass systems (Biosharp, BS‐15‐RC) and cultured for 24 h under standard conditions (37°C, 5% CO_2_). For SCAP Golgi trafficking analysis, cells were fixed in pre‐chilled (‐20°C) ethanol for 10 min at RT, followed by three 5 min PBS washes. After permeabilization with 0.2% Triton X‐100 (5 min) and blocking with 5% BSA (1 h), samples were incubated overnight at 4°C with primary antibodies: anti‐SCAP (1:500; Proteintech, 12266‐1‐AP) and anti‐Golgin 97 (1:500; CST, 97537S).

SREBP2 nuclear translocation studies employed 4% PFA followed by identical permeabilization and blocking procedures. Samples were probed with anti‐SREBP2 (1:500; Santa Cruz, sc‐13552) overnight at 4°C. All samples subsequently received appropriate fluorescent‐dye‐conjugated secondary antibodies and DAPI counterstaining.

Cholesterol‐ER colocalization was assessed by incubating live cells with 1 µm ER‐Tracker Green (Beyotime, C1042M) in serum‐free DMEM (37°C, 30 min). Following three washes with pre‐warmed serum‐free medium, cells were briefly fixed in 4% PFA (2 min, 37°C) and stained with 50 µg mL^−1^ Filipin III (30 min, light‐protected). Nuclear counterstaining used PI. Fluorescence images were captured using a Nikon A1R HD25 confocal microscope. Fluorescence intensity was quantified using ImageJ with consistent thresholding parameters across samples.

### 4.11 Lipid ROS Measurement

MKN1 and NUGC4 cells were harvested and subjected to three consecutive centrifugation cycles (1000 × rpm, 4 min, 4°C) with PBS washes. The cell pellet was resuspended in 1 mL PBS containing 10 µm C11‐BODIPY 581/591 probe (Thermo Fisher Scientific, D3861) and incubated for 10 min at 37°C under 5% CO_2_. Post‐staining, the cells were subjected to three additional PBS wash‐centrifugation cycles (1000 × rpm, 4 min). The final cell suspension (500 µL in PBS) was filtered through a 40‐µm cell strainer (Falcon, 352340) to remove aggregates. Flow cytometric analysis was performed on a CytoFLEX LX flow cytometer (Beckman Coulter). A minimum of 10,000 viable cells per sample was acquired in low‐speed acquisition mode using the FITC channel for oxidized BODIPY detection. Data was analyzed using Flowjo v10.4.0 software. Gating strategies excluded debris and doublets based on forward/side scatter profiles and pulse width discrimination.

### 4.12 Malondialdehyde (MDA) Content Assay

Cell lysates were prepared by homogenizing 1 × 10^6^ cells in 100 µL ice‐cold Western & IP Cell Lysis Buffer (Beyotime Biotechnology, P0013) supplemented with 1× protease inhibitor cocktail (Biosharp, BL1426A). Lysates were clarified by centrifugation (12 000 × *g*, 10 min, 4°C) and total protein concentrations were determined using the BCA Protein Assay Kit (Beyotime Biotechnology, P0011) to normalize malondialdehyde (MDA) levels. MDA quantification was performed using the Lipid Peroxidation MDA Assay Kit (Beyotime Biotechnology, S0131) according to the manufacturer's specifications. Briefly, 100 µL of supernatant was reacted with a 0–200 µm MDA standard curve in microcentrifuge tubes. Samples were incubated with 200 µL MDA detection working solution containing thiobarbituric acid (TBA) at 100°C for 15 min using a water bath. Following cooling to RT in a water bath, the reaction mixtures were centrifuged at 1000 × *g* for 10 min to remove precipitates. The chromogenic supernatant (200 µL) was transferred to a 96‐well flat‐bottomed plate, and the absorbance was measured at 532 nm using a Spark multimode microplate reader (Tecan Spark Devices).

### 4.13 Measurement of Intracellular Ferrous Iron (Fe^2^^+^) Levels

Cellular Fe^2^^+^ concentrations were quantified using the cell ferrous iron (Fe^2+^) fluorometric assay kit (Elabscience, E‐BC‐F101) according to the manufacturer's recommended protocols. Briefly, 1 × 106 cells were harvested and subjected to two consecutive centrifugation cycles (300 × *g*, 5 min, 4°C) with wash buffer Reagent 1. The cell pellet was resuspended in 1 mL Reagent 2 buffer containing 5 µm Fe^2+^ probe and incubated for 30 min at 37°C under 5% CO_2_ in the dark. Post‐staining, the cells were subjected to three additional wash‐centrifugation cycles in Reagent 1 buffer (300 × *g*, 5 min), and the final cell suspension (500 µL in Reagent 1) was filtered through a 40 µm cell strainer (Falcon, 352340) to remove aggregates. Flow cytometric analysis was performed on a CytoFLEX LX flow cytometer (Beckman Coulter). A minimum of 10,000 viable cells per sample was acquired in low‐speed acquisition mode using the PE channel for Fe^2+^ detection. Data was analyzed using Flowjo v10.4.0 software. Gating strategies excluded debris and doublets based on forward/side scatter profiles and pulse width discrimination.

### 4.14 Gastric Cancer Organoid Culture and Drug Treatment

Fresh tumor samples were washed with DPBS containing 100 U mL^−1^ penicillin‑‐streptomycin until clear. Tissues were transferred into a digestion solution containing collagenase type I (Sigma‑Aldrich, V900891), type II (Gibco, 17101015), and type IV (Worthington, LS004188) in Advanced DMEM/F12 (Gibco, 12634028), and incubated in a 37°C water bath for 45 min with shaking every 10 minutes until no clumps remained. Digests were filtered (70 µm), centrifuged (300 × *g*, 5 min), and the pellets resuspended in a 1:1 mixture of gastric organoid culture medium (Advanced DMEM/F12 with 10 mm HEPES (Gibco, 15630130), 1 × GlutaMAX (Gibco, 35050061), 100 U mL^−1^ penicillin‑streptomycin, and Matrigel (Corning, 354277). Additional supplements included 50% WNT‑, 10% R‑spondin‑, and 10% Noggin‑conditioned media, together with 1.25 mm N‑acetyl‑L‑cysteine (Sigma‐Aldrich, A9165), 200 ng mL^−1^ FGF10 (PeproTech, 100–26), and 50 ng mL^−1^ EGF (Gibco, PHG0313). Mixtures (100 µL per well) were seeded into 12‑well plates and allowed to solidify (1 h, 37 °C, 5% CO_2_), then 1 mL culture medium was added. Medium was renewed every 4 d. For passaging, Matrigel was dissolved in Advanced DMEM/F12 with 10 mm HEPES, 100 U mL^−1^ penicillin streptomycin, and 1 × GlutaMAX. Organoids were collected, centrifuged, and then dissociated with pre‑warmed TrypLETM (Gibco, 12563029) under monitoring with pipetting every 5 min until single cells were obtained. Cells were reseeded as above. Organoids were passaged once per week.

For drug treatment, after digestion and counting, organoids were resuspended in Matrigel, and 100 µL droplets were seeded into 12‑well plates and allowed to solidify. Then, 1 mL of complete medium containing the test drug (control: 0.1% DMSO) was added per well. After 48 h, the medium was replaced with fresh drug‑containing medium. Incubation continued until organoids reached an appropriate size. Daily monitoring and bright‑field imaging recorded morphological changes (shrinkage, blebbing, loss of budding, debris).

### 4.15 Statistical Analysis

Statistical analyses were performed using GraphPad Prism v9.5.1. Differences between two groups were assessed by the unpaired Student's t‐test. Multiple group comparisons were analyzed by one‐ or two‐way ANOVA followed by Tukey's post hoc test. Survival curves were compared using the log‐rank (Mantel‐Cox) test. Differential expression of genes, proteins, and pathways was evaluated using appropriate R packages. Results are presented as the means ± SDs., and statistical significance was defined as a *p* < 0.05.

## Author Contributions

J.‐J.Q., Q.X., G.P., and W.Z. conceived this study. J.‐J.Q., G.P., W.Z., F.T., and Q.H. supervised the study. Q.X., G.P., L.Z., X.C., Z.D., A.C., H.S., and Y.Z. performed bioinformatics data analysis and molecular and cell biology experiments. Q.X. and G.P. performed SCAP functional experiments in xenograft and orthotopic mouse models and the IHC and HE staining of tissue samples from GC patients. K.Z. conducted organoid model experiments. X.C. collected the clinical samples for this study. Y.L. performed experiments in molecular dynamics simulations and molecular docking. Q.X., G.P., L.Z., H.J., H.X., Y.Z., X.D., K.M., Q.H., F.T., W.Z., and J.‐J.Q. wrote and revised the manuscript, and all co‐authors reviewed the manuscript.

## Funding

This work was supported by the National Natural Science Foundation of China (No. 82203275), Natural Science Foundation of Zhejiang Province (No. QKHM25H3103, LRG26H160002), Natural Science Foundation of Hangzhou (No. 2025SZRJJ2312), “Pioneer” and “Leading Goose” R&D Program of Zhejiang (No. 2024SDYXS0003), Program of Zhejiang Provincial TCM Sci‐Tech Plan (No. GZY‐ZJ‐KJ‐24064 & No. 2025ZR099), NSFC Regional Innovation and Development Joint Fund (U25A20613), and CACMS Innovation Fund (No. CI2023C012LH).

## Ethics Statement

All animal experiments were approved by the Animal Ethics Committee of the Hangzhou Institute of Medicine (HIM), Chinese Academy of Sciences (AP2025‐12‐0670). All clinical samples were approved by the Research Ethics Committees of Zhejiang Cancer Hospital (MR‐33‐25‐090737).

## Conflicts of Interest

The authors declare no conflicts of interest.

## Supporting information




**Supporting File**: advs76290‐sup‐0001‐SuppMat.doc.

## Data Availability

The mass spectrometry proteomics data have been deposited to the ProteomeXchange Consortium (https://proteomecentral.proteomexchange.org) via the iProX partner repository [66, 67], with the dataset identifier PXD069338. The RNA‐seq data utilized in this study is available in the Genome Sequence Archive for Human (https://ngdc.cncb.ac.cn/gsa‐human/policy/policy.jsp) under accession codes HRA014319. All raw data and any additional information generated in this study are available from the lead contact upon a reasonable request.
