# Supplementary material for: Functional Suppression of SCAP Triggers Endoplasmic Reticulum Stress‐Dependent Ferroptosis by Impairing Cholesterol Metabolism in Gastric Cancer
Source: Adv Sci (Weinh). 2026 Jun 29:e76290. Online ahead of print. doi: 10.1002/advs.76290 (PMC13336778; doi:10.1002/advs.76290)
Supplement: Supplementary file 1 — Supporting File: advs76290‐sup‐0001‐SuppMat.doc. [file ADVS-9999-e76290-s001.doc]

*Supporting Information*

**Functional Suppression of SCAP Triggers Endoplasmic Reticulum Stress-Dependent Ferroptosis by Impairing Cholesterol Metabolism in Gastric Cancer**

Qianqian Xu^1, 3, 4, 13^, Guangzhao Pan^1,3,13^*, Lele Zhang^5,13^, Xiangliu Chen^3,13^, Kui Zhang^6^, Yang Lu^7^, Zibo Duan^1^, Aiping Chen^8^, Hailong Shen^1^, Yuxi Zhang^1^, Xiaowu Dong^7^, Jinzhou Huang^3^, Xing Huang^9^, Kai Miao^10^, Qian Hua^7^*, Fangfang Tao^4,11^*, Weidong Zhang^2,12^*, Jiang-Jiang Qin^1,3,14^*

**Contents**

**Supplementary Materials and Methods**

**Supplemental Figures**

FIGURE S1. Analysis of SCAP expression characteristics in GC patients.

FIGURE S2. Identification of functional inhibitors targeting the SCAP SSD.

FIGURE S3. PD activates *de novo* cholesterol synthesis and impairs cholesterol efflux.

FIGURE S4. PD-mediated SREBP2 activation depends on the SCAP SSD.

FIGURE S5. PD triggers Nrf1 nuclear translocation via cholesterol-dependent ER stress.

FIGURE S6. PD promotes ER stress-dependent ferroptosis.

FIGURE S7. PD suppresses GC tumor growth *in vivo* via ER stress-dependent ferroptosis without obvious organs toxicity.

**Supplemental Tables**

Supplemental Table S1. Key reagents and resources table

Supplemental Table S2. Antibody information table

Supplemental Table S3. Sequences of primers used for RT-qPCR

Supplemental Table S4. The targeting sequences for knockdown of GFP and SCAP

**Reference**

**Supplementary Materials and Methods**

**Cell lines**

The HEK293T human embryonic kidney cell line (RRID: CVCL_0063; ATCC CRL-3216) was obtained from the American Type Culture Collection (ATCC, USA). Human GC cell lines MKN1 (RRID: CVCL_1415) and NUGC4 (RRID: CVCL_3082) were purchased from Cobioer Biosciences Co., Ltd. (Nanjing, China). MKN1 and NUGC4 cells were maintained in RPMI-1640 medium supplemented with 10% fetal bovine serum (FBS) and 1% penicillin-streptomycin (P/S). HEK293T cells were cultured in DMEM with 10% FBS and 1% P/S. The NUGC4‑luc derivative and SCAP‑knockdown stable lines were grown in the same base medium additionally containing 50 μg/mL hygromycin B or 4 μg/mL puromycin, respectively.

**Plasmids**

For stable SCAP knockdown, short hairpin RNA (shRNA) sequences were designed using the BROAD Institute’s GPP portal (https://portals.broadinstitute.org/gpp/public/seq/search) and cloned into the pLKO.1 vector. Lentiviral particles were produced by co-transfecting the constructed plasmid with packaging plasmids pLP1, pLP2, and pVSV-G. The SCAP vectors plasmid (pLVX-AcGFP1-N1) was purchased from YouBio (Changsha, China). All constructs were verified by sequencing prior to use (See **Supplemental Table S1 for details**).

**Immunohistochemistry (IHC) and hematoxylin-eosin (H&E) staining**

Formalin-fixed, paraffin-embedded (FFPE) tumor tissues (2 µm sections) were processed using standardized immunohistochemistry protocols ^1^. Sections underwent deparaffinization in xylene and graded ethanol rehydration (100% to 70%). Antigen retrieval was performed by microwave heating in preheated 10 mM sodium citrate buffer at 95-98 °C for 10 minutes, followed by 30 minutes cooling at RT. Endogenous peroxidase activity was quenched with 3% H₂O₂ (10 minutes, RT), and nonspecific binding was blocked using 1 × Animal-Free Blocking Solution (Cell Signaling Technology, 15019; 30 minutes, RT). After three 5 minutes PBS washes, sections were incubated overnight at 4 °C with primary antibodies (SCAP, 1:300, Proteintech; SREBP2, 1:50, Invitrogen) in a humidified chamber. Following PBS rinses, slides were treated with biotinylated goat anti-rabbit IgG and horseradish peroxidase-conjugated streptavidin for 1 hour, followed by imaging with a microscope.

For H&E staining, tissue samples were fixed in 4% paraformaldehyde (PFA) (24 hours, 4 °C), dehydrated through graded ethanol series (70%, 80%, 95%, 100%), cleared in xylene, and embedded in paraffin. Sections (2 μm thick) were stained using a commercial H&E kit (Art. ZLI-9609 ZSGB-BIO Corp., Shanghai, China) according to the manufacturer's protocols: hematoxylin immersion for 5 minutes, eosin counterstaining for 1 minute, and graded ethanol dehydration. Representative images of five fields per sample were captured under a microscope.

**Virtual screening workflow and molecular docking**

The virtual screening workflow initiated with the procurement of a library of 4260 commercially available compounds from our laboratory. Primary filtering by Lipinski's Rule of Five (RO5) criteria eliminated 1,279 compounds lacking favorable drug-like properties, yielding 2,981 candidates with optimal pharmacokinetic profiles. Subsequently, molecular docking was performed for the top 50 compounds using the Glide module (Schrödinger Suite, version 2021-3) with standard precision (SP) mode, and the 10 top-ranked ligands based on docking scores and binding mode analysis were prioritized for further experimental validation.

Molecular docking was then performed using the Schrödinger Suite (version 2021-3) through an integrated workflow. Briefly, the crystal structures of SCAP (PDBID: 7ETW) were retrieved from the RCSB protein database and removes the A-chain and other ligand molecular structures from 7ETW, retaining only the B-chain SCAP protein portion, and then modifies the missing heavy atoms in the SCAP protein using PDBFixer 1.10. Small molecule ligands, including Platycodin D (PubChem CID: 162859), were downloaded in SDF format from the PubChem Compound Database. Molecular docking was implemented as binding pocket docking using AutoDock Vina1.2.5 software, and we used DoGSiteScorer for binding pocket position prediction and sorted the predicted binding pockets according to DrugScore. Pymol and Discovery Studio Visualizer software (version 24.1.0) were utilized to analyze and visualize the forces in 2D and 3D angles.

**Molecular dynamics simulation**

Molecular Dynamics (MD) simulations analyze dynamic structural changes in molecular recognition (e.g., ligand-protein interactions) by integrating structural flexibility and environmental factors. This enhances binding conformation sampling, refines affinity predictions, and identifies structural cavities for optimized drug design, surpassing rigid docking methods in biological realism and accuracy. MD serves as a bridge between static structural data and functional biomolecular behavior, addressing critical limitations in traditional computational drug discovery pipelines.

All-atom MD simulations were performed using GROMACS 2021 under periodic boundary conditions. The AMBER99SB force field was employed for both protein and ligand parameterization. The PD-SCAP complex was solvated with TIP3P water molecules in a cubic box constructed using GROMACS utilities, with system neutrality achieved through Na⁺/Cl⁻ ion addition (0.15 M final concentration). All simulations were performed at 298.15 K with a production run duration of 200 ns. Energy minimization was performed using the steepest descent algorithm with a convergence criterion of maximum force < 1000 kJ·mol⁻¹·nm⁻¹. Non-bonded interactions were treated with a 1.2 nm cutoff for both Coulombic and van der Waals forces. System equilibration included variable temperature (NVT, 100 ps) and isothermal-isobaric ensemble (NPT, 100 ps) phases, implemented using the V-rescale thermostat (τ=0.1 ps) and Parrinello-Rahman barostat (τ = 2.0 ps), respectively. Root Mean Square Deviation (RMSD) of Cα atoms relative to the initial conformation was calculated to assess global structural stability. Radius of Gyration (Rg) quantified protein compactness through mass-weighted atomic distances. Root Mean Square Fluctuation (RMSF) mapped per residue flexibility using backbone atom positional variances. Hydrogen bond occupancy and secondary structure persistence were additionally monitored using DSSP algorithms. All analyses employed time-averaged values from the equilibrated trajectory (50-200 ns).

**RNA extraction and quantitative real-time PCR**

After three consecutive washes with PBS, MKN1 and NUGC4 cells were harvested and lysed. Total RNA was prepared using a FastPure® Cell/Tissue Total RNA Isolation Kit (Vazyme Biotech). RNA integrity and concentration were verified using NanoDrop One (Thermo Fisher Scientific), with all samples showing A260/A280 ratios between 1.8-2.0. First-strand cDNA synthesis was performed using the Fast All-in-One RT Kit according to the manufacturer's specifications. Quantitative PCR amplification was performed with 2 × Taq Pro Universal SYBR qPCR Master Mix (Vazyme Biotech) on a CFX96 Touch Real-Time PCR Detection System. All reactions were performed in technical triplicates to ensure statistical robustness. Relative mRNA quantification was achieved through the comparative threshold cycle (2^-ΔΔCT^) method, with normalization to endogenous GAPDH expression levels. Sequences of primers used for PCR were listed in the **Supplemental Table S3**.

**Transmission electron microscopy (TEM) assay**

After experimental treatments, MKN1 and NUGC4 cells were scraped and pelleted by centrifugation at 1,000 × rpm for 4 minutes. Cell fixation was performed sequentially: primary fixation in 2.5% glutaraldehyde (Biosharp, BL911A) at 4 °C for 6 hours, followed by post-fixation with 1% osmium tetroxide in the same buffer for 1 hour at 4 °C. Afterward, dehydration was carried out through a graded ethanol series (30%, 50%, 70%, 80%,90%, and 100%) with three exchanges of anhydrous acetone. Samples were infiltrated and embedded in SPI-Pon 812 epoxy resin (Electron Microscopy Sciences, Hatfield, PA, USA). Ultrathin sections (50-70 nm) were prepared on a Leica EM UC7 ultramicrotome (Leica Microsystems, Buffalo Grove, IL, USA). Sections were double-contrasted with 2% uranyl acetate (30 minutes) and lead citrate (15 minutes). TEM imaging was performed on a JEM-2100 Plus field emission gun microscope (JEOL Ltd., Tokyo, Japan) operating at 120 kV.

**Lentiviral knockdown and stable cell line generation**

Lentiviral vectors encoding shRNA targeting human SCAP (lenti-shSCAP) and control (lenti-shGFP) were designed using an online tool from the BROAD Institute. The sequences of the shRNAs (5’-3’) used were listed in the **Supplemental Table S4**. Lentiviral particles were generated using shRNA targeting human SCAP plasmids (shSCAP 1#, 2# and 3#) and packaging vectors (pLP1, pLP2 and pVSV-G) in HEK293T cells. Briefly, plasmid DNA was mixed with Lipofectamine 2000 in 500 μL Opti-MEM (Gibco, 31985070) and incubated for 20 minutes at RT. HEK293T cells (2 × 10^6^ cells/well, 80-90% confluence) in 6-well plates were transfected with the mixture. The culture medium was replaced with fresh DMEM 8 hours after transfection. Virus-containing supernatants were harvested at 48 hours post-transfection and filtered through a 0.45 μm filter membrane. Aliquots were stored at -80 °C.

For stable cell line generation, NUGC4 cells at 30-50% confluence was transduced with lentiviral particles in the presence of 10 μg/mL polybrene for 48 hours. Post-transduction media was replaced with complete growth medium containing 8 μg/mL puromycin for 2-4 weeks to select positively transduced cells. Cells were maintained in medium containing 4 μg/mL puromycin to establish stable knockdown cell lines. Knockdown efficiency was validated by Immunoblotting using anti-SCAP antibody (Abcam, ab125186; 1:1,000) normalized to Actin (Beyotime Biotechnology, AF0003; 1:1,000).

**Proteome and transcriptome sequencing analysis**

For RNA sequencing (RNA-seq), 5 × 10⁶ MKN1 cells were seeded in 10-cm dishes and treated with PD (10 μM) or vehicle control for 24 hours (The selection of drug concentrations in this study refers to our previously published article ^2^). Total RNA was extracted using TRIzol™ reagent (Thermo Fisher, 15596018) according to the manufacturer's instructions. RNA quantity and integrity were evaluated using an Agilent 2100 Bioanalyzer with the RNA Nano 6000 Assay Kit (Agilent Technologies, CA, USA). Paired-end sequencing (150 bp) was performed on an Illumina NovaSeq 6000 (Shanghai Applied Protein Technology Co., Ltd., China) following standard protocols.

For proteomic analysis, 8 × 10⁶ MKN1 cells were seeded in 10-cm dishes and treated with PD (10 μM) or vehicle control for 24 hours. After that, the cells were rinsed with PBS to remove serum and harvested by scraping on ice. The data independent acquisition (DIA) method was used to quantify total protein levels. For protein extraction and digestion, the proteins were extracted using an SDT buffer (4% SDS, 100 mM Tris-HCl, pH 7.6) and quantified using the BCA method. The samples were then reduced with 40 mM DTT (37 °C, 600 × rpm, 1.5 hours), alkylated with 20 mM IAA (30 minutes, dark), and digested with trypsin (1:50 w/w, 37 °C, 15-18 hours) using 10 kDa centrifugal filters. The peptides were desalted with C18 cartridges C18 Cartridges (Empore™ SPE Cartridges MCX, 30UM, waters), concentrated, and reconstituted in 0.1% formic acid. The Peptides were spiked with indexed retention time (iRT) standards and analyzed on an Orbitrap™ Astral™ mass spectrometer (MS) (Shanghai Applied Protein Technology Co., Ltd., China) coupled to an Vanquish Neo system liquid chromatography (Thermo Scientific) in DIA mode.

Full MS scans (380-980 m/z) were acquired at 240,000 resolution (200 m/z), with MS/MS spectra collected in 299 DIA windows (2 m/z isolation, 25 eV HCD collision energy). Data were processed using DIA-NN (v1.8.1) with trypsin/P digestion parameters, carbamidomethyl (C) as fixed modification, oxidation (M) and acetyl (N-term) as variable modifications, and a 1% false discovery rate (FDR) threshold.

Supplemental Figures


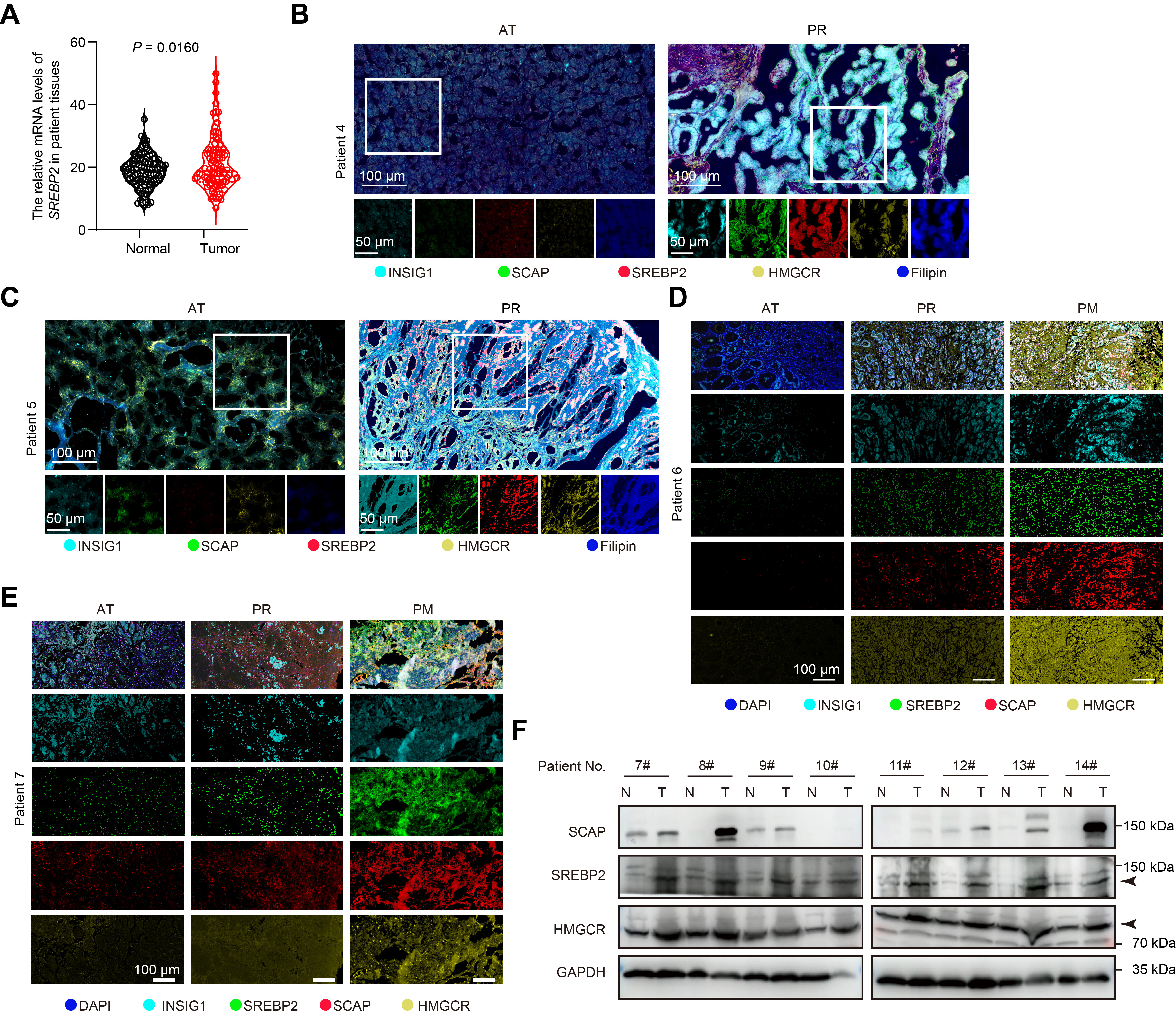


**FIGURE S1.** Analysis of SCAP expression characteristics in GC patients. **(A)** *SREBP2* mRNA levels in paired GC tissues (n = 75). **(B, C)** Representative multiplex immunohistochemical images showing SCAP, SREBP2, HMGCR and Filipin staining in paired GC tissues from patient 4 (**B**) and Patient 5 (**C**). AT, normal adjacent tissues; PR, primary tumor tissue. Scale bar = 100 μm. **(D, E)** Representative images showing SCAP, SREBP2, HMGCR, and INSIG1 expression across different GC pathological subtypes in patient 6 (**D**) and Patient 7 (**E**). PM, primary metastatic tissue. Scale bar = 100 μm. **(F)** Immunoblots of SCAP, SREBP2, and HMGCR in eight paired GC patient samples (Patients 8-15). N, normal tissue; T, tumor tissue.

**
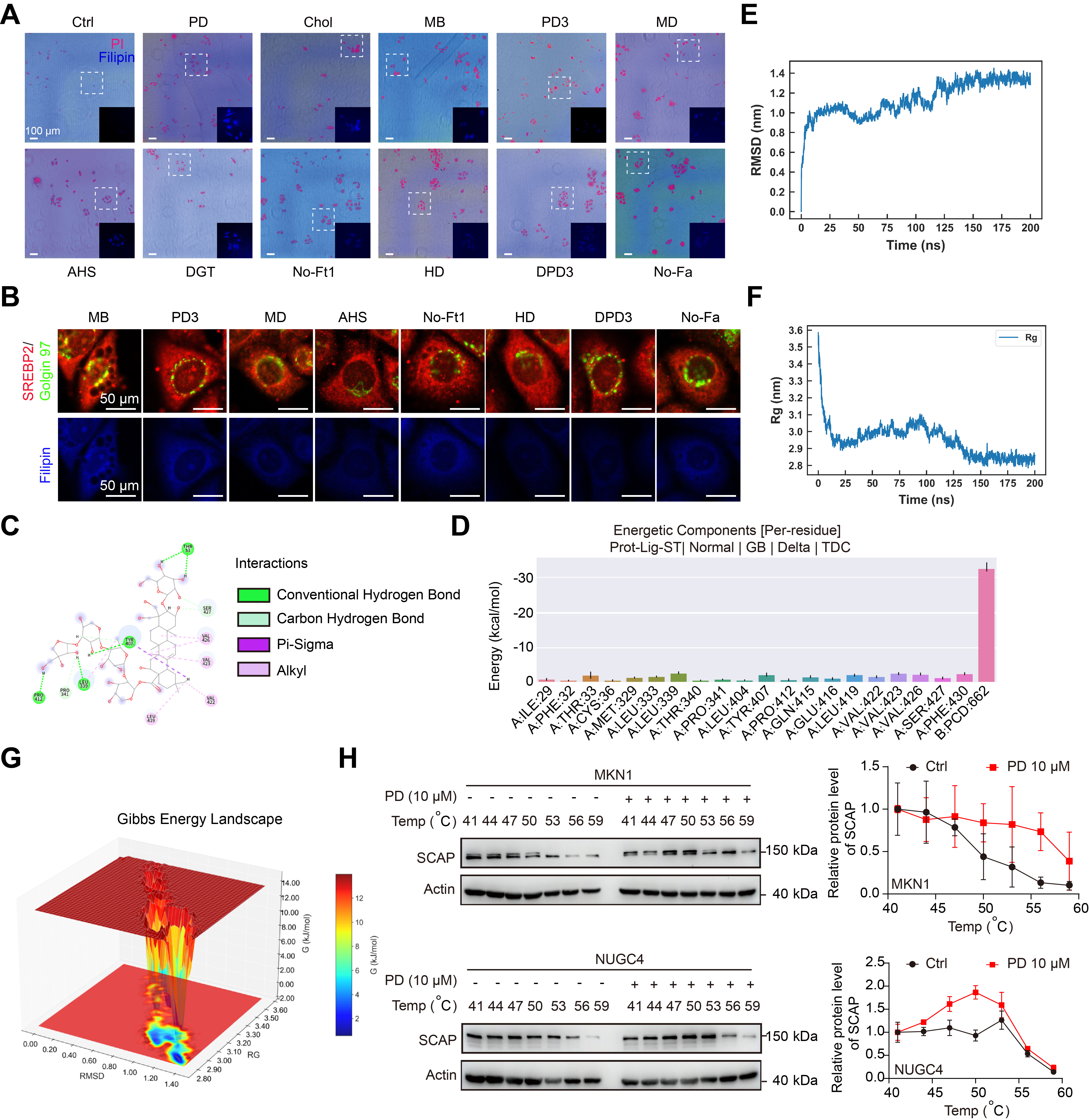
**

**FIGURE S2.** Identification of functional inhibitors targeting the SCAP SSD. **(A)** Representative high-content images of intracellular cholesterol (Filipin staining) in cells treated with the top 10 candidate compounds. Nuclei were stained with propidium iodide (PI). Ctrl, control; PD, platycodin D; Chol, cholesterol; MB, macranthoside B; PD3, platycodin D3; MD, madecassoside; AHS, anemarrhenasaponin I；DGT, digitonin; No-Ft1, notoginsenoside Ft1; HD, hederacoside D; DPD3, deapi-platycodin D3; No-Fa, notoginsenoside Fa. Scale bar = 100 μm. **(B)** Representative immunofluorescence images showing SREBP2 colocalization with the Golgi (marked by Golgin 97) and intracellular cholesterol (Filipin staining) in cells treated with indicated compounds. Scale bar = 50 μm. **(C)** Two-dimensional diagram of interactions between the SCAP SSD and PD. Green dashed lines: hydrogen bonds; dashed light green lines: carbon-hydrogen bonds; dashed purple lines: Pi-Sigma interactions; dashed pale pink lines: alkyl hydrophobic interactions. **(D)** Key amino acid residues mediating SCAP SSD interactions. **(E, F)** Root means square deviation (RMSD) (**E**) and Radius of Gyration (Rg) (**F**) analysis of the SCAP SSD-PD complex during simulation. **(G)** Three-dimensional Gibbs free energy landscapes illustrating conformational energy distribution of the SCAP-PD complex. **(H)** Immunoblots showing the effect of PD on thermal stability of SCAP in MKN1 and NUGC4 cells. The curve charts show quantified protein levels. Temp, temperature.

**
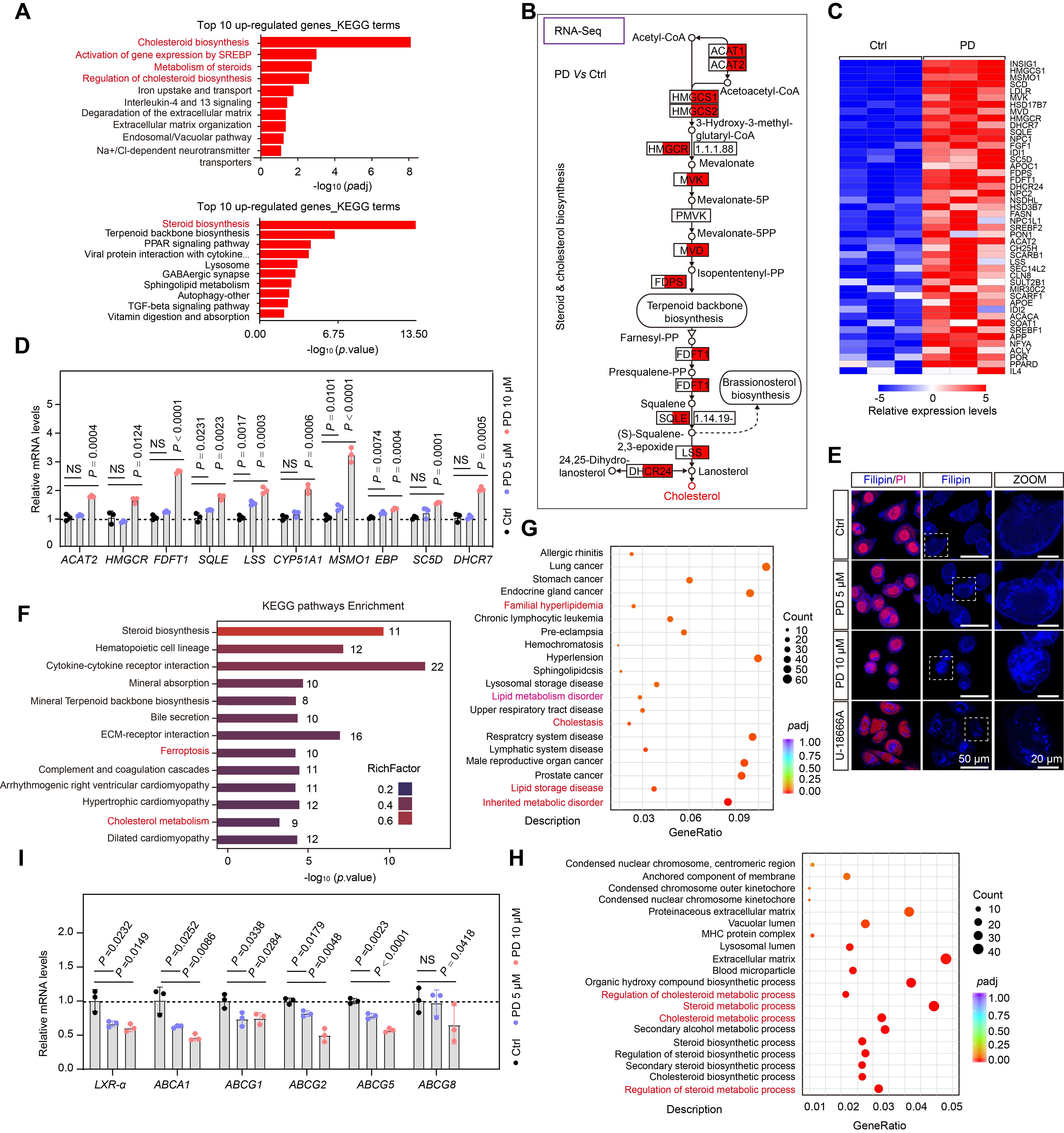
**

**FIGURE S3.** PD activates *de novo* cholesterol synthesis and impairs cholesterol efflux. **(A)** KEGG pathway analysis of the top 10 upregulated genes in PD- versus vehicle-treated cells (RNA-seq, top; Proteomics, bottom). **(B)** Pathway analysis of steroid/cholesterol biosynthesis based on RNA-seq data, showing mRNA enrichment in PD-treated versus control cells. Red rectangles indicate upregulation; gray indicates no change (n = 3 cell replicates per group). **(C)** Heatmap of differentially expressed cholesterol synthesis-related mRNAs from RNA-seq data in PD- versus vehicle-treated cells. **(D)** Relative mRNA levels of steroid/cholesterol biosynthesis genes in vehicle- and PD-treated NUGC4 cells (n=3 biological replicates). **(E)** Representative immunofluorescence images of intracellular cholesterol (Filipin staining) in cells treated with vehicle, PD (5 μM or 10 μM), or U-18666A. Original scale bar = 50 μm; zoom scale bar = 20 μm. **(F)** KEGG pathway enrichment analysis of differentially expressed proteins from proteomics data. **(G, H)** Bubble plot of significantly enriched pathways from RNA-seq data by KEGG (**G**) and GO (**H**) analyses. **(I)** Relative mRNA levels of cholesterol efflux genes in vehicle- and PD-treated NUGC4 cells (n = 3 biological replicates). NS, not significant.

**
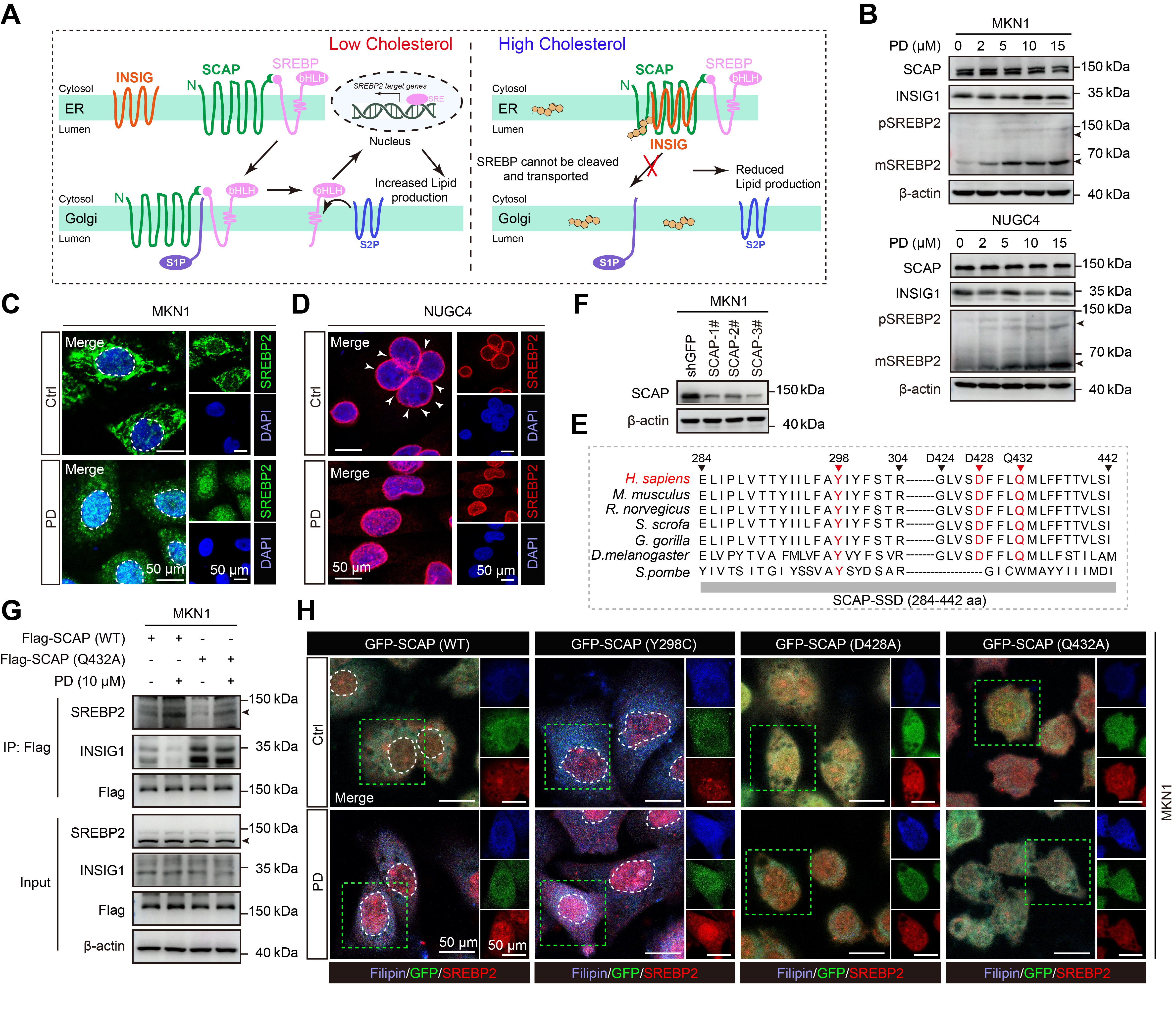
**

**FIGURE S4.** PD-mediated SREBP2 activation depends on the SCAP SSD. **(A)** Schematic of cellular cholesterol homeostasis regulation. Low cholesterol promotes SCAP-SREBP2 interaction and *de novo* biosynthesis; high cholesterol enhances SCAP-INSIG1 binding and feedback inhibition. ER, endoplasmic reticulum; S1P, site-1 proteases; S2P, site-2 proteases; bHLH, basic helix-loop-helix. **(B)** Immunoblots of SCAP, INSIG1, and SREBP2 in MKN1 and NUGC4 cells treated with 0-15 μM PD for 24 hours. **(C, D)** Representative immunofluorescence images of SREBP2 nuclear translocation in MKN1 (**C**) and NUGC4 (**D**) cells treated with vehicle or 10 μM PD for 24 hours. Scale bar = 50 μm. **(E)** Conservation analysis of SCAP amino acids 284-442 across species. **(F)** Immunoblots of SCAP in MKN1 cells transfected with GFP or SCAP 1#, 2#, and 3# knockdown plasmids. **(G)** Immunoblots of Flag, SREBP2, and INSIG1 in SCAP-knockdown MKN1 cells reconstituted with Flag-SCAP WT or Q432A mutant after PD treatment and Flag immunoprecipitation. **(H)** Representative immunofluorescence images of intracellular cholesterol (Filipin staining) in SCAP-knockdown MKN1 cells expressing WT or mutant Flag-SCAP (Y298C, D428A, Q432A), treated with vehicle or 10 μM PD for 24 hours. White dashed circle indicates nuclei. Scale bar = 50 μm.


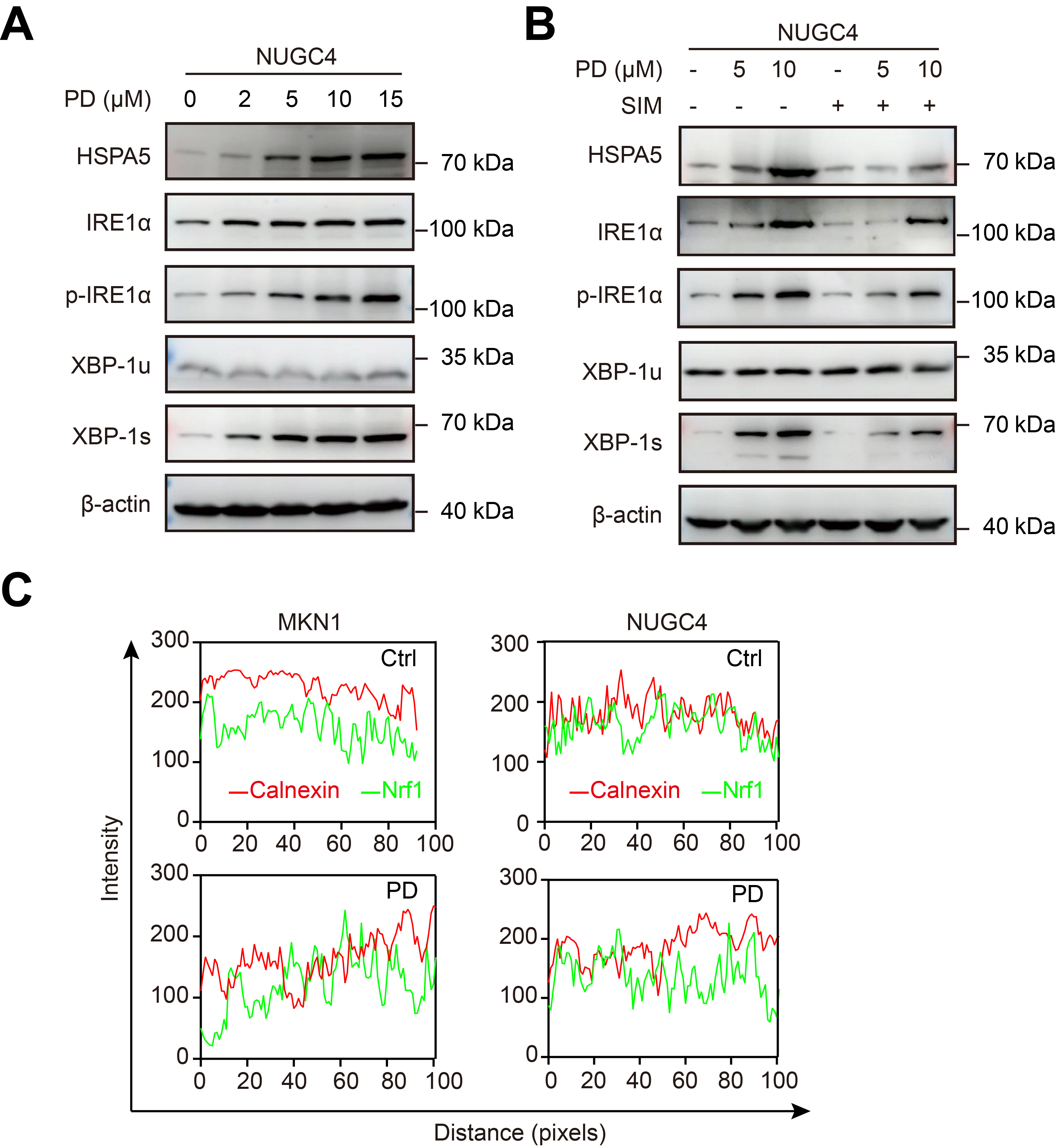


**FIGURE S5.** PD triggers Nrf1 nuclear translocation via cholesterol-dependent ER stress. **(A, B)** Immunoblots of IRE1α/XBP-1 pathway proteins in NUGC4 cells treated with vehicle, PD alone (**A**), or PD combined with simvastatin (SIM) (**B**). **(C)** Quantification of Nrf1-ER colocalization in MKN1 and NUGC4 cells. Colocalization was analyzed in predefined regions using ImageJ. The ER was marked by calnexin (See **Figure 5F** for additional information).

**
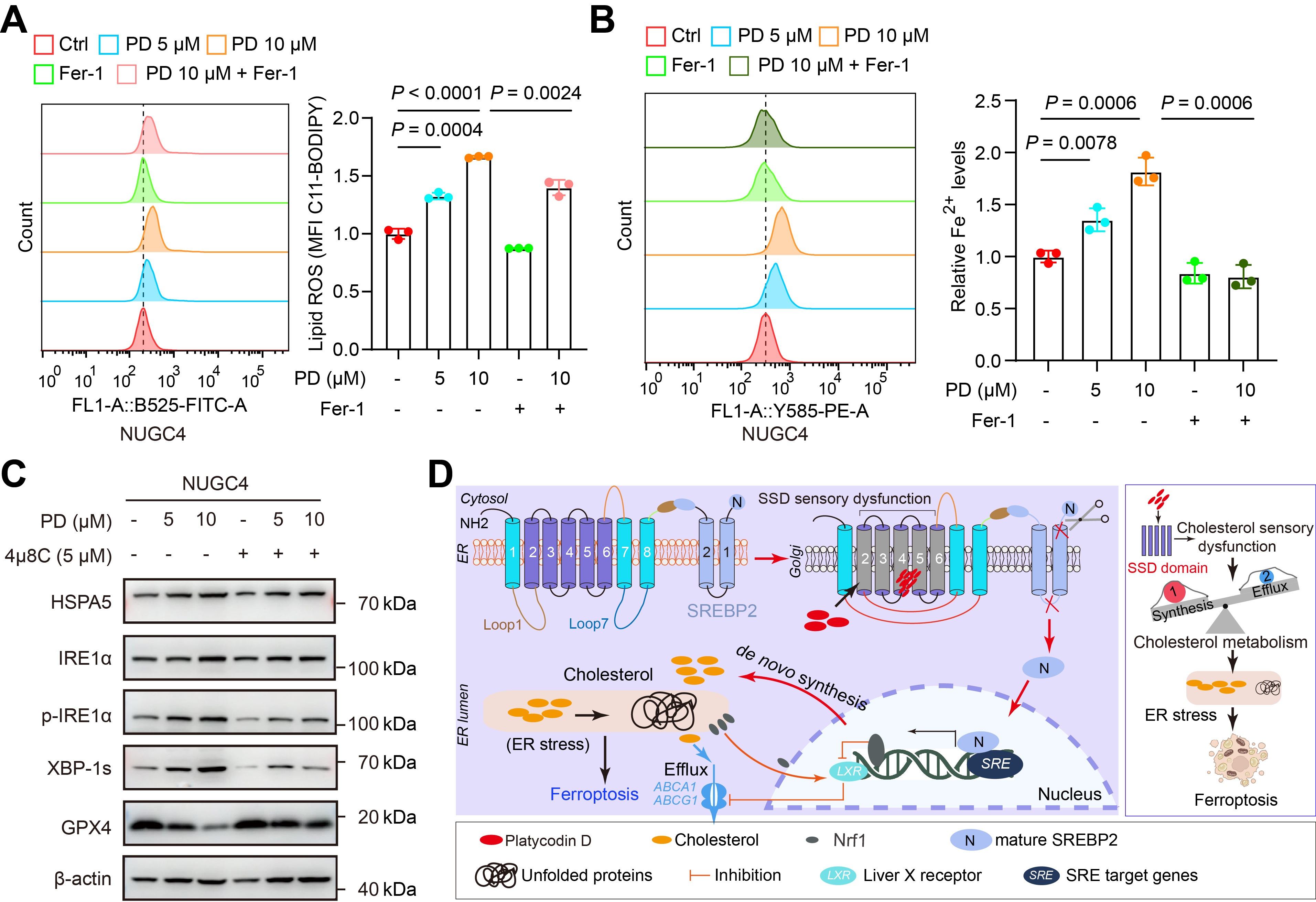
**

**FIGURE S6.** PD promotes ER stress-dependent ferroptosis. **(A)** Representative flow cytometry plots and quantification of lipid ROS levels in NUGC4 cells treated with vehicle, PD alone, or PD combined with 5 μM ferrostatin-1 (Fer-1) (n = 3 biological replicates). **(B)** Representative flow cytometry plots and quantification of Fe^2+^ levels in NUGC4 cells under the same treatment conditions as in (**A**) (n = 3 biological replicates). **(C)** Immunoblots of IRE1α/XBP-1 pathway activation and GPX4 expression in NUGC4 cells treated with vehicle, PD alone, or PD combined with the IRE1α inhibitor 4μ8C. **(D)** Proposed model: PD targets the SCAP SSD, disrupts cholesterol metabolism, and induces ER stress-dependent ferroptosis in GC.

**
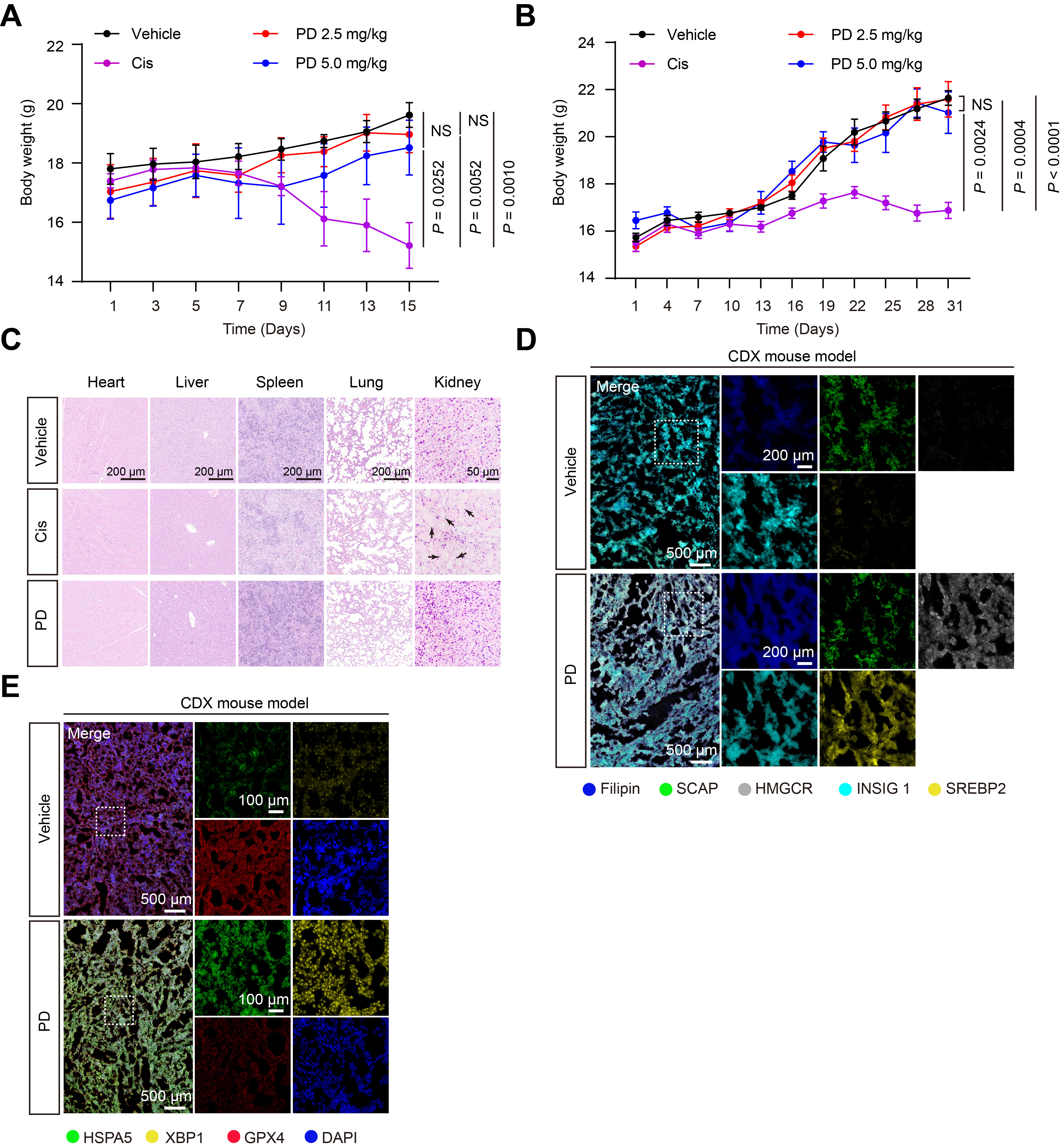
**

**FIGURE S7.** PD suppresses GC tumor growth *in vivo* via ER stress-dependent ferroptosis without obvious organs toxicity. **(A， B)** Body weight curves of mice in the CDX (**A**) and PDX (**B**) models treated with vehicle, PD (2.5 or 5.0 mg/kg), or cisplatin (Cis, 1.5 mg/kg). CDX, cell line-derived xenograft; PDX, patient-derived xenograft. Data are presented as mean ± SEM. **(C)** Histological analysis of major organs (heart, liver, spleen, lung, kidney) from PDX-bearing mice after treatment with vehicle, PD (2.5 or 5.0 mg/kg), or Cis (1.5 mg/kg). Arrow indicates renal damage in the Cis group. Scale bar: 200 μm (heart, liver, spleen, lungs); 50 μm (kidney). **(D)** Representative multiplex immunohistochemical images showing SCAP, HMGCR, INSIG1, SREBP2, and Filipin staining in CDX tumor tissues. Scale bar = 500 μm. **(E)** Representative multiplex immunohistochemical images showing HSPA5, XBP-1, and GPX4 in CDX tumor tissues. Scale bar = 500 μm. NS, not significant.

**Supplementary Tables**

**Supplemental Table S1. Key reagents and resources table**

| **Reagents Source Lot NO.** | | |
| --- | --- | --- |
| **Critical commercial assays** | | |
| EndoFree Maxi Plasmid Kit | TIANGEN | DP117 |
| Hematoxylin-eosin (H&E) staining kit | ZSGB-BIO | ZLI-9609 |
| BCA Protein Assay Kit | Beyotime | P0011 |
| Cell Counting Kit-8 (CCK-8) | Beyotime | C0037 |
| Amplex Red Cholesterol and Cholesteryl Ester Assay Kit | Beyotime | S0211S |
| ER-Tracker Green | Beyotime | C1042M |
| FastPure® Cell/Tissue Total RNA Isolation Kit V2 | Vazyme | RC113-01 |
| Taq Pro Universal SYBR qPCR Master Mix | Vazyme | Q712-02 |
| Fast All-in-One RT Kit (with gDNA Remover) | ESScience | ES-RT001 |
| Cell Ferrous Iron (Fe2+) Fluorometric Assay Kit | Elabscience | E-BC-F101 |
| Lipid Peroxidation MDA Assay Kit | Beyotime | S0131S |
| **Reagents** | **Source** | **Lot NO.** |
| Platycodin D | Chengdu Must Bio-technology Co., Ltd | 58479-68-8 |
| Notoginsenoside Fa | Chengdu Must Bio-technology Co., Ltd | 88100-04-3 |
| Deapi-platycodin D3 | Chengdu Must Bio-technology Co., Ltd | 67884-05-3 |
| Hederacoside D | Chengdu Must Bio-technology Co., Ltd | 760961-03-3 |
| Notoginsenoside Ft1 | Chengdu Must Bio-technology Co., Ltd | 155683-00-4 |
| Anemarrhenasaponin I | Chengdu Must Bio-technology Co., Ltd | 163047-21-0 |
| Madecassoside | Chengdu Must Bio-technology Co., Ltd | 34540-22-2 |
| Platycodin D3 | Chengdu Must Bio-technology Co., Ltd | 67884-03-1 |
| Macranthoside B | Chengdu Must Bio-technology Co., Ltd | 146100-02-9 |
| Cholesterol (Water Soluble) | MCE | HY-N0322A |
| Digitonin | MCE | HY-N4000 |
| Filipin | MCE | HY-N6716 |
| U-18666A | MCE | HY-107433 |
| Simvastatin | MCE | HY-17502 |
| Ferrostatin-1 | MCE | HY-100579 |
| Cisplatin | MCE | HY-17394 |
| Tunicamycin | MCE | HY-A0098 |
| 4μ8C | MCE | HY-19707 |
| Hieff Trans® Polyethylenimine Linear (PEI) MW40000 | Yeasen | 40816ES02 |
| D-Luciferin,Potassium Salt | Yeasen | 40902ES01 |
| 2.5% Glutaraldehyde Fixative | Biosharp | BL911A |
| Antifade Mounting Medium  with DAPI | Beyotime | P0131 |
| DAPI | Beyotime | C1005 |
| Propidium Iodide | Yeasen | 40711ES10 |
| 4% Paraformaldehyde Fixative | Biosharp | BL539A |
| Triton X-100 | Beyotime | ST795 |
| BSA | Sangon Biotech (Shanghai) Co., Ltd | 9048-46-8 |
| Goat Serum | Beyotime | C0265 |
| Lipofectamine 2000 | Invitrogen | 11668-019 |
| Polybrene | Beyotime | ST1380 |
| Puromycin | MCE | HY-K1057 |
| BODIPY™ 581/591 C11 | Thermo fisher Scientific | D3861 |
| ECL Chemiluminescent Substrate | Biosharp | BL520B |
| PMSF | Biosharp | BL1426A |
| PhosSTOP™ | Roche | 04906837001 |
| RIPA Lysis Buffer | Beyotime | P0013B |
| Western & IP Cell Lysis Buffer | Beyotime | P0013 |
| Protein A+G Agarose | Beyotime | P2055 |
| Collagenase type I | Sigma‑Aldrich | V900891 |
| Collagenase type II | Gibco | 17101015 |
| Collagenase type IV | Worthington | LS004188 |
| Advanced DMEM/F12 | Gibco | 12634028 |
| HEPES | Gibco | 15630130 |
| GlutaMAX™ | Gibco | 35050061 |
| Matrigel | Corning | 354277 |
| N‑acetyl‑L‑cysteine | Sigma-Aldrich | A9165 |
| FGF10 | PeproTech | 100-26 |
| EGF | Gibco | PHG0313 |
| TrypLE^TM^ | Gibco | 12563029 |
| **Recombinant DNA** | **Source** | **Lot NO.** |
| pLKO.1 Vector | Addgene | 10878 |
| pLKO.1: GFP | This paper | N/A |
| pLKO.1: SCAP KD1 | This paper | N/A |
| pLKO.1: SCAP KD2 | This paper | N/A |
| pLKO.1: SCAP KD3 | This paper | N/A |
| pLP1 | Addgene | 209988 |
| pLP2 | Addgene | 209989 |
| VSV-G | Addgene | 8454 |
| SCAP pLVX-AcGFP1-N1 | Youbio | 58922 |
| SCAP D428A 3xFlag pLVX-AcGFP1-N1 | Youbio | L24882 |
| SCAP Y298C 3xFlag pLVX-AcGFP1-N1 | Youbio | L24883 |
| SCAP Q432A 3xFlag pLVX-AcGFP1-N1 | Youbio | L24884 |
| SCAP Δ280-450AA 3xFlag pLVX-AcGFP1-N1 | Youbio | L24885 |
| SCAP-HIS PCDH-PURO | Youbio | 57251 |
| SCAP-HIS (280-450AA) PCDH-PURO | Youbio | L24881 |
| pLVX-CMV Vector | This paper | N/A |
| pCDH-CMV Vector | Addgene | 72265 |
| **Software** |  |  |
| Microsoft Excel 2019 | Microsoft | https://www.microsoft.com/zh-cn |
| Primer Premier 5.0 | Premier Biosoft | http://www.premierbiosoft.com/index.html |
| CaseViewer | N/A | https://www.3dhistech.com/solutions/caseviewer/ |
| GraphPad Prism 9.5.1 | GraphPad | https://www.graphpad.com/ |
| ImageJ | N/A | https://github.com/imagej |
| Living Image 4.4 | N/A | https://living-image.software.informer.com/4.1/ |
| Photoshop | Photoshop Biosoft | https://www.photoshop.com/ |
| Adobe Illustrator CS5 | Adobe Biosoft | https://www.adobe.com/ |
| GSEA v2.0.14 | N/A | https://www.gsea-msigdb.org/gsea/index.jsp |
| Flowjo 10.4.0 | FlowJo | https://www.FlowJo.com/ |
| BioRender | BioRender | https://www.biorender.com/ |

**Supplemental Table S2. Antibody information table**

| **Antibodies** | **Source** | **Lot NO.** | **Dilution Ratio** |
| --- | --- | --- | --- |
| Rabbit anti-SCAP | Proteintech | 12266-1-AP | 1:250 (IP),1:250 (IHC) |
| Rabbit anti-SCAP | Abcam | ab125186 | 1:1000 (WB),1:100 (IP) |
| Rabbit anti-SCAP | Abcam | ab190103 | 1:1000 (WB),1:100 (IF),1:200 (IHC) |
| Mouse anti-SREBP2 | Santa Cruz | sc-271616 | 1:1000 (WB),1:250 (IF),1:250 (IHC) |
| Rabbit anti-SREBP2 | Invitrogen | PA5-24167 | 1:1000 (WB),1:50 (IF),1:50 (IHC) |
| Mouse anti-β-Actin | Beyotime | AF0003 | 1:1000 (WB) |
| Mouse anti-Flag | Thermo fisher Scientific | MA1-91878 | 1:1000 (WB) |
| Mouse anti-Flag | Sigma-Aldrich | F1804 | 1:1000 (WB) |
| Mouse anti-His | Beyotime | AF2876 | 1:1000 (WB) |
| Rabbit anti-His | CST | #12698 | 1:1000 (WB) |
| Mouse anti-GFP | Beyotime | AF2882 | 1:1000 (WB) |
| Mouse anti-INSIG1 | Santa Cruz | sc-390504 | 1:1000 (WB),1:250 (IP),1:250 (IHC) |
| Mouse anti-HMGCR | Santa Cruz | sc-271595 | 1:1000 (WB),1:250 (IHC) |
| Rabbit anti-HMGCR | HUABIO | ET1702-41 | 1:1000 (WB),1:100 (IHC) |
| Rabbit anti-GPX4 | CST | #52455S | 1:1000 (WB) |
| Rabbit anti-GPX4 | Abcam | ab125066 | 1:1000 (WB),1:200 (IHC) |
| Rabbit anti-IRE1α | CST | #3294S | 1:1000 (WB) |
| Rabbit anti-p-IRE1α | Abcam | ab48187 | 1:1000 (WB),1:300 (IHC) |
| Rabbit anti-XBP1 | CST | #12782S | 1:1000 (WB) |
| Rabbit anti-XBP1 | Abcam | Ab109221 | 1:1000 (WB),1:250 (IHC) |
| Rabbit anti-HSPA5 | CST | #3177S | 1:1000 (WB),1:250 (IHC) |
| Mouse anti-HSPA5 | Proteintech | 66574-1-Ig | 1:1000 (WB),1:1000 (IHC) |
| Mouse anti-Golgin-97 | CST | #97537S | 1:1000 (WB),1:200 (IF) |
| Rabbit anti-Histone H3 | CST | #4499 | 1:1000 (WB) |
| Mouse anti-GAPDH | CST | #97166 | 1:1000 (WB) |
| Mouse anti-Calnexin | Santa Cruz | sc-23954 | 1:1000 (WB),1:200 (IF) |
| Rabbit anti-NFE2L1 | Thermo fisher Scientific | PA5-90023 | 1:1000 (WB),1:100 (IF) |
| Mouse anti-NFE2L1 | HUABIO | RT1440 | 1:1000 (WB),1:250 (IF) |
| Rabbit anti-IgG | Beyotime | A7016 | 1:100 (IP) |
| Mouse anti-IgG | Beyotime | A7028 | 1:100 (IP) |
| Anti-mouse IgG, HRP-linked | CST | #7076S | 1:1000 (WB) |
| Anti-rabbit IgG, HRP-linked | CST | #7074S | 1:1000 (WB) |
| F(ab')2-Goat anti-Rabbit IgG (H+L) Cross-Adsorbed Secondary Antibody, Alexa Fluor™ 594 | Invitrogen | A-11072 | 1:1000 (IF) |
| F(ab')2-Goat anti-Rabbit IgG (H+L) Cross-Adsorbed Secondary Antibody, Alexa Fluor™ 488 | Invitrogen | A-11070 | 1:1000 (IF) |
| F(ab')2-Goat anti-Mouse IgG (H+L) Cross-Adsorbed Secondary Antibody, Alexa Fluor™ 488 | Invitrogen | A-11017 | 1:1000 (IF) |
| F(ab')2-Goat anti-Mouse IgG (H+L) Cross-Adsorbed Secondary Antibody, Alexa Fluor™ 594 | Invitrogen | A-11020 | 1:1000 (IF) |

**Supplemental Table S3. Sequences of primers used for RT-qPCR**

| **Gene Name** | **Forward Primer Sequence**  **(5' to 3')** | | **Reverse Primer Sequence**  **(5' to 3')** |
| --- | --- | --- | --- |
| GAPDH | GGAGCGAGATCCCTCCAAAAT | GGCTGTTGTCATACTTCTCATGG | |
| ACAT2 | TGGTGCCTTAGCTGCTGTTCCT | GGCTTGTCTAACAGGATTCTGCC | |
| HMGCR | AGGAGGCATTTGACAGCACT | ACCTGGACTGGAAACGGATA | |
| FDFT1 | ATAACCAATGCACTGCACCA | CCTTTCCGAATCTTCACTGC | |
| SQLE | ACCCGAGTCCAGTTCTCATCTA | CCTTGGCATTTCTCCTCTAATG | |
| LSS | GCACTGGACGGGTGATTATGG | TCTCTTCTCTGTATCCGGCTG | |
| CYP51A1 | GCTCAGTTGTTCCCTGCTTC | AAAATTAGCCAGGCATGGTG | |
| MSMO1 | TGCTTTGGTTGTGCAGTCATT | GGATGTGCATATTCAGCTTCCA | |
| EBP | CTCAGCACCTAAGACTGGACA | ACGACTAAGACCCCTGTGACA | |
| SC5D | ACCATACGTGTATCCAGCCAC | GCTCAGTGTTGCACAGAAGAAA | |
| DHCR7 | GCTGCAAAATCGCAACCCAA | GCTCGCCAGTGAAAACCAGT | |
| LXR-α | GTTATAACCGGGAAGACTTTGCCA | GCCTCTCTACCTGGAGCTGGT | |
| ABCA1 | GCACTGAGGAAGATGCTGAAA | AGTTCCTGGAAGGTCTTGTTCAC | |
| ABCG1 | CAGGAAGATTAGACACTGTGG | GAAAGGGGAATGGAGAGAAGA | |
| ABCG2 | ACGAACGGATTAACAGGGTCA | CTCCAGACACACCACGGAT | |
| ABCG5 | ACTGCTTCTCCTACGTCCTG | CTGTAGTTGCCAATCAGTCGG | |
| ABCG8 | AGCCTCCTTGCTAGATGTGAT | GTCTCTCGCACAGTCAAGTTG | |

**Supplemental Table S4. The targeting sequences for knockdown of GFP and SCAP**

| **Gene name** | **Sequences (5’-3’)** |
| --- | --- |
| GFP Forward | CCGGGAATTAGATGGTGATGTTAATCTCGAGATTAACATCACCATCTAATTCTTTTTG |
| GFP Reverse | AATTCAAAAAGAATTAGATGGTGATGTTAATCTCGAGATTAACATCACCATCTAATTC |
| SCAP 1#-Forward | CCGGCCGACGCTCTTCAGCTATTACCTCGAGGTAATAGCTGAAGAGCGTCGGTTTTTG |
| SCAP 1#-Reverse | AATTCAAAAACCGACGCTCTTCAGCTATTACCTCGAGGTAATAGCTGAAGAGCGTCGG |
| SCAP 2#-Forward | CCGGGCTCAACGGTTCCCTTGATTTCTCGAGAAATCAAGGGAACCGTTGAGCTTTTTG |
| SCAP 2#-Reverse | AATTCAAAAAGCTCAACGGTTCCCTTGATTTCTCGAGAAATCAAGGGAACCGTTGAGC |
| SCAP 3#-Forward | CCGGTGCTTAATTGACACCAACTTTCTCGAGAAAGTTGGTGTCAATTAAGCATTTTTG |
| SCAP 3#-Reverse | AATTCAAAAATGCTTAATTGACACCAACTTTCTCGAGAAAGTTGGTGTCAATTAAGCA |

**Reference**

1. Li S, Yuan L, Xu ZY, et al. Integrative proteomic characterization of adenocarcinoma of esophagogastric junction. *Nat Commun.* Feb 11 2023;14(1):778.

2. Xu Q, Pan G, Wang Z, et al. Platycodin-D exerts its anti-cancer effect by promoting c-Myc protein ubiquitination and degradation in gastric cancer. *Front Pharmacol.* 2023;14:1138658.
